# A new species of *Cordyligaster* Macquart, reared from caterpillars in Area de Conservacion Guanacaste, northwestern Costa Rica

**DOI:** 10.3897/BDJ.2.e4174

**Published:** 2014-11-24

**Authors:** AJ Fleming, D Monty Wood, M Alex Smith, Daniel Janzen, Winnie Hallwachs

**Affiliations:** † Canadian National Collection, Ottawa, Canada; ‡ Department of Integrative Biology, University of Guelph, Guelph, Canada; § Department of Biology, University of Pennsylvania, Philadelphia, Philadelphia, United States of America

**Keywords:** *Cordyligaster*, Diptera, Tachinidae, tropical rain forest, tropical dry forest, parasitoid fly, host-specificity, caterpillar inventory, Crambidae

## Abstract

We describe a new species of *Cordyligaster* Macquart (Diptera: Tachinidae) from Area de Conservacion Guanacaste (ACG) in northwestern Costa Rica. *Cordyligaster capellii* **sp. n.**, is described and photographed. All specimens of *C. capellii* were reared from *Syngamia florella* (Stoll, 1781) (Lepidoptera, Crambidae, Spilomelinae), a leaf-rolling caterpillar collected in ACG rain forest. By coupling morphology, photographic documentation, life history and molecular data, we provide a clear and concise description of this new species. In addition the authors provide new distribution and host records for *C. fuscipennis* (Macquart) reared in ACG.

## Introduction

*Cordyligaster* Macquart, 1844, is a small new world genus of the subfamily Dexiinae (Diptera, Tachinidae, Dexiinae). This genus is easily distinguished from other Dexiine genera of tachinids by its narrow and petiolate abdomen (Figs 1, 2, 5, 6).  Townsend 1936, placed *Cordyligaster* within the tribe Sophiini, a most likely polyphyletic tribe including the following genera: *Euantha* Wulp, *Euanthoides* Townsend, *Leptidosophia* Townsend, *Neosophia* Guimarães, *Neoeuantha* Townsend, *Sophia* Robineau-Desvoidy, and *Sophiella* Guimarães. Guimarães 1982, offered the following diagnosis of the tribe Sophiini: body, legs, wings, and abdomen elongate; antenna arising below midline of eye with long plumose aristae; legs, hind tibiae with reduced posteroventral bristle.

The last major systematic treatment of this genus was Guimarães 1971, where 5 species of *Cordyligaster* were reported. Sabrosky 1973 published a further restudy of Guimarães’ efforts, where he proposed a few changes namely the clarification of differences between *C. petiolata*, and *C. fuscipennis*, two names which had long been held in synonymy. Wood and Zumbado (2010) mention 7 known species within *Cordyligaster* from Central and South America, with only one of them having been reared from Costa Rica; it was believed (in error) to be *Cordyligaster petiolata* (Wiedemann) and we discuss that here. Our aims are to build further on this knowledge base by providing a new rearing record of *C. fuscipennis* from ACG, and describing an additional new species, for this little known genus. All specimens studied here, were obtained from leaf-rolling Crambidae caterpillars collected in ACG, in northwestern Costa Rica. The new species described herein was recognized as new by differences in external morphology, male terminalia, and CO1 (coxI or cytochrome oxidase 1) gene sequences. The data gleaned on parasitoid biology and associated hosts from ACG efforts is invaluable in providing definitive information on the biology, and associated hosts of the reared parasitoids species. These are the first rearing records for the genus *Cordyligaster* within Costa Rica. Despite the exhaustive nature of the ACG inventory, there may well be more species of *Cordyligaster* in ACG and Costa Rica, yet to be discovered.

## Materials and methods

### Acronyms for depositories

CNC              Canadian National Collection of Insects, Arachnids and Nematodes, Ottawa, Canada

USNM           National Museum of Natural History, Washington, D.C., USA

INBio            Instituto Nacional de Biodiversidad, Santo Domingo de Heredia, Costa Rica

SMF              Forschungsinstitut und Naturmuseum Senckenberg, Frankfurt-am-Main, Germany

MZSP            Museu de Zoologia Universidade de São Paulo, São Paulo, Brazil

### Geographic area of the study and rearing intensity

All flies and rearing information described here were found by the 35+ year–old ongoing inventory of the caterpillars, their food plants and their parasitoids of the dry forest, rain forest, cloud forest, and intergrades, in the 125,000+ ha terrestrial portion of Area de Conservación Guanacaste (ACG) in northwestern Costa Rica (Rodriguez et al. 2012, Janzen and Hallwachs 2011, Janzen et al. 2009, Smith et al. 2007, Smith et al. 2006, Smith et al. 2008, Smith et al. 2012, Smith et al. 2009, Fernández-Triana et al. 2014). The tachinid rearing methods are described at http://janzen.bio.upenn.edu/caterpillars/methodology/how/parasitoid_husbandry.htm. In brief, caterpillars (and sometimes pupae) are found in the wild at all instars by a wide variety of search methods, and reared in captivity on the food plant species on which they were found, until they produced an adult, a parasitoid, or died of other causes. Each caterpillar is documented as an individual, the emerging parasitoid flies are first treated as a rearing event and later as individuals once they are barcoded.

In the near 4 decades since its inception in 1978, this inventory has reared approximately 600,000 wild-caught caterpillars. All frequencies of parasitization reported here must be considered against the background of this inventory. Equally, it is patently obvious that although the inventory is carried out throughout the year, there is a bias towards certain environments, types of vegetation and distance off the ground. Comparison of reared species of parasitoids with those collected by net or Malaise traps demonstrates that to date, the caterpillar inventory has so far encountered well less than half the species of caterpillar parasitoids present in ACG. The largest unsampled void is the upper foliage of the canopy above about 3-4 m above the ground.

The treatment reported here is focused on placing names on the species reared, thereby preparing them for later detailed ecological and behavioral accounts and studies that will normally extend across ACG ecological groups, whole ecosystems, and taxonomic assemblages much larger than a genus.

DNA barcodes (standardised 5’ region of the mitochondrial cytochrome *c* oxidase I (COI) gene) for all ACG inventory specimens were obtained using DNA extracts prepared from single legs using a glass fibre protocol (Ivanova et al. 2006). Total genomic DNA was re-suspended in 30 μl of dH_2_O, and a 658-bp region near the 5’ terminus of the CO1 gene was amplified using standard primers (LepF1–LepR1) following established protocols (Smith et al. 2007, Smith et al. 2006, Smith et al. 2008). All information for the sequences associated with each individual specimen (including GenBank and BOLD accession) can be retrieved from the Barcode of Life Data System (BOLD) (Ratnasingham and Hebert 2007) via the publically available dataset: http://dx.doi.org/10.5883/DS-ASCORDY [DOI requested 141015 - not yet active].

### Imaging and Dissections

Our descriptions of new species are deliberately brief and only include some differentiating descriptions of body parts and colors that are commonly used in Tachinid identification. These brief descriptions are complemented with an extensive series of color photos of every species to illustrate the readily observed differences among these species.

Habitus photographs were taken using a Canon T3i digital SLR, using a 65mm Macro Photo Lens 1:2.8 (MP–E 65mm), mounted on a microscope track stand (AmScope, Model: TS200) modified to accept a Manfrotto QR 200PL–14 quick release plate. Images were shot in aperture priority, allowing the camera to control shutter speed at f/4.5, over 40 images at equal distance increments. Illumination was provided with a homemade reflective dome (instruction for dome creation can be found at: http://www.cdfa.ca.gov/plant/ppd/entomology/Dome/kd–200.html) placed over a 144 LED ringlight (AmScope, Model: LED–144–YK).

Adult fly dissections followed standard practice (O’Hara 1983). Photographs of male terminalia were taken using a Canon S110 digital camera adaptor mounted to the eyepiece of a Leitz–Wetzlar dissecting scope. Preparations were mounted on a depression slide in a small quantity of Rexall brand hand sanitizer gel (NPN# 80007138). This allowed the specimen to remain steady in a given position, and therefore provided a better environment for photography. After mounting and photographing, the terminalia were rinsed in a small quantity of pure distilled water, before being replaced in the glycerine–filled microvial.

The photographic series were created using Photoshop CS6, and Zerene Stacker Software v1.04. So as to maximize image quality and depth of field, photo series were digitally stacked to produce a final composite image.

The terminology used for genitalia (which refers here only to the sclerotized parts of the genitalia, and not to the soft internal structures) and other body parts follows Cumming and Wood (2009).

All specimens listed as examined are considered paratypes, except for the holotype, which is noted separately.

### Voucher specimen management

All caterpillars reared from ACG efforts receive a unique voucher code in the format of yy–SRNP–xxxxx. Any parasitoid emerging from this caterpillar receives the same voucher code, and then if/when later the parasitoid is dealt with individually, it receives a second voucher code unique to it, in the format of DHJPARxxxxxxx. The voucher codes and collateral data assigned to both host and emergent parasitoids are available at http://janzen.bio.upenn.edu/caterpillars/database.lasso. To date, all DHJPARxxxxxxx coded tachinids have had one leg removed for attempted DNA barcoding at the Biodiversity Institute of Ontario (BIO) in the University of Guelph, with all collateral data and all successful barcodes permanently and publically deposited in the Barcode of Life Data System (BOLD, www.boldsystems.org) (Suppl. material 2), and later migrated to GenBank as well. A neighbor–joining (NJ) tree (Saitou and Nei 1987) for all *Cordyligaster* reared and DNA barcoded by this inventory through 2013 is included as Suppl. material 1. The inventory grows continually and new specimens can be found by searching the genus *Cordyligaster* in BOLD. Each barcoded specimen also has an accession code in the Barcode of Life Data System (BOLD) and GenBank.

Inventoried Tachinidae were collected under Costa Rican government research permits issued to DHJ since 1978, and likewise exported under permit by DHJ from Costa Rica to Philadelphia, and then to the final depository in the Canadian National Insect collection in Ottawa, Canada. Tachinid identifications for the inventory were done by DHJ in coordination with a) visual inspection by AJF and DMW, b) DNA barcoding by BIO, MAS, and BOLD, and c) correlation with host caterpillar identifications by DHJ and WH through the inventory itself. Dates of capture of each reared fly in the inventory are the dates of eclosion of the fly, and not the date of capture of the caterpillar. This is because the fly eclosion date is much more representative of the time when that fly species is on the wing than is the time of capture of the caterpillar or (rarely) finding a parasitized pupa. However, the collector listed is the parataxonomist who found the caterpillar, rather than the person who retrieved the newly eclosed fly from its rearing bag or bottle, and processed it by freezing, pinning, labeling and oven–drying. Fly biology and degrees of parasitization by these flies will be the detailed subject of later papers.

### Generic Synonyms of *Cordyligaster*

*Cordyligaster* Macquart 1844: 90. Type species: *Dexia petiolata* Wiedemann, 1830, by original designation.

Cordylidexia Giglio-Tos 1894: 67. (unecessary *nomen novum* for *Cordyligaster* Macquart). Type species: *Dexia petiolata* Wiedemann, 1830, by original designation.

*Megistogaster* Macquart 1851: 185. Type species: *Megistogaster fuscipennis* Macquart, 1851, by original designation.

*Eucordylidexia* Townsend 1915: 41. Type species: *Eucordylidexia ategulata* Townsend, 1915 (=*Dexia petiolata* Wiedemann, 1830), by original designation.

*Eucordyligaster* Townsend 1917: 123. Type species: *Cordyligaster septentrionalis* Townsend, 1909, by original designation.

### Species included in *Cordyligaster*

*analis* Macquart 1851: 187 (*Megistogaster*). Holotype male (BMNH) [examined by DMW]. Type locality: Brazil, Amazonas.

*fuscipennis* Macquart 1851: 186. Syntypes, 3 males (BMNH) [examined by DMW]. Type locality: South America (as Java in error). Resurrected from synonymy by (Sabrosky 1973: 221).

*ategulata* Townsend 1915: 41. Holotype female (USNM) [examined by DMW]. Type locality: Guatemala, Puerto Barrios. Synonymy by (Sabrosky 1973: 221).

*minuscula* van der Wulp 1891: 252. Syntypes 10 males, 8 females (BMNH) [examined by DMW]. Type localities: (1 male) Mexico, Guerrero, Rio Papagaio; (6 males, 4 females) Tierra Colorado 2000 feet; (3 males, 4 females) Tabasco, Teapa.

*septentrionalis* Townsend 1909: 250. Holotype female (USNM) [examined by DMW]. Type locality: USA, Maryland, Plummers Island. Synonymy established by (Thompson 1910: 212).

*nyomala* Townsend 1914: 93. Holotype male (USNM) [examined by DMW]. Type locality: Peru, Upper Piura Valley, Ñomala.

*petiolata* Wiedemann 1830: 374 (Dexia). Holotype male (SMF) [examined by DMW]. Type locality: Brazil.

*fuscifacies* Bigot 1888: 101. Holotype female (BMNH) [examined by DMW]. Type locality: "Java" [in error], presumably South America.

*tipuliformis* Walker 1858: 205. Holotype male (BMNH) [examined by DMW]. Type locality: South America.

*townsendi* Guimarães 1971: 101. Holotype male (MZSP).Type locality: Brazil, Mato Grosso, Pôsto Garapu, Rio Sete de Setembro.

## Taxon treatments

### Cordyligaster capellii

Fleming & Wood, 2014 sp. n.

urn:lsid:zoobank.org:act:23389AF1-D6FD-4455-AD01-33640EEE6C76

#### Materials

- **Type status:** Holotype. **Occurrence:** occurrenceDetails: http://janzen.sas.upenn.edu; catalogNumber: DHJPAR0006938; recordedBy: D.H. Janzen & W. Hallwachs, Manuel Rios; individualID: DHJPAR0006938; individualCount: 1; sex: male; lifeStage: adult; preparations: pinned; otherCatalogNumbers: 06-SRNP-30780,ASTAV180-06; **Taxon:** scientificName: Cordyligaster capelli; phylum: Arthropoda; class: Insecta; order: Diptera; family: Tachinidae; genus: Cordyligaster; specificEpithet: capelli; scientificNameAuthorship: Fleming & Wood; **Location:** continent: Central America; country: Costa Rica; countryCode: CR; stateProvince: Guanacaste; county: Sector Pitilla; locality: Area de Conservacion Guanacaste; verbatimLocality: Amonias; verbatimElevation: 390; verbatimLatitude: 11.04249; verbatimLongitude: -85.40339; verbatimCoordinateSystem: Decimal; decimalLatitude: 11.04249; decimalLongitude: -85.40339; **Identification:** identifiedBy: AJ Fleming; dateIdentified: 2014; **Event:** samplingProtocol: Host Collection; verbatimEventDate: 20-Feb-2006; **Record Level:** language: en; institutionCode: CNC; collectionCode: Insects; basisOfRecord: Pinned Specimen
- **Type status:** Paratype. **Occurrence:** occurrenceDetails: http://janzen.sas.upenn.edu; catalogNumber: DHJPAR0006939; recordedBy: D.H. Janzen & W. Hallwachs, Manuel Rios; individualID: DHJPAR0006939; individualCount: 1; lifeStage: adult; preparations: pinned; otherCatalogNumbers: 06-SRNP-30016,ASTAV181-06; **Taxon:** scientificName: Cordyligaster capelli; phylum: Arthropoda; class: Insecta; order: Diptera; family: Tachinidae; genus: Cordyligaster; specificEpithet: capelli; scientificNameAuthorship: Fleming & Wood; **Location:** continent: Central America; country: Costa Rica; countryCode: CR; stateProvince: Guanacaste; county: Sector Pitilla; locality: Area de Conservacion Guanacaste; verbatimLocality: Pasmopa; verbatimElevation: 440; verbatimLatitude: 11.019; verbatimLongitude: -85.41; verbatimCoordinateSystem: Decimal; decimalLatitude: 11.019; decimalLongitude: -85.41; **Identification:** identifiedBy: AJ Fleming; dateIdentified: 2014; **Event:** samplingProtocol: Host Collection; verbatimEventDate: 23-Jan-2006; **Record Level:** language: en; institutionCode: CNC; collectionCode: Insects; basisOfRecord: Pinned Specimen
- **Type status:** Paratype. **Occurrence:** occurrenceDetails: http://janzen.sas.upenn.edu; catalogNumber: DHJPAR0006940; recordedBy: D.H. Janzen & W. Hallwachs, Manuel Rios; individualID: DHJPAR0006940; individualCount: 1; lifeStage: adult; preparations: pinned; otherCatalogNumbers: 06-SRNP-30179,ASTAV182-06; **Taxon:** scientificName: Cordyligaster capelli; phylum: Arthropoda; class: Insecta; order: Diptera; family: Tachinidae; genus: Cordyligaster; specificEpithet: capelli; scientificNameAuthorship: Fleming & Wood; **Location:** continent: Central America; country: Costa Rica; countryCode: CR; stateProvince: Guanacaste; county: Sector Pitilla; locality: Area de Conservacion Guanacaste; verbatimLocality: Pasmopa; verbatimElevation: 440; verbatimLatitude: 11.019; verbatimLongitude: -85.41; verbatimCoordinateSystem: Decimal; decimalLatitude: 11.019; decimalLongitude: -85.41; **Identification:** identifiedBy: AJ Fleming; dateIdentified: 2014; **Event:** samplingProtocol: Host Collection; verbatimEventDate: 02-Feb-2006; **Record Level:** language: en; institutionCode: CNC; collectionCode: Insects; basisOfRecord: Pinned Specimen
- **Type status:** Paratype. **Occurrence:** occurrenceDetails: http://janzen.sas.upenn.edu; catalogNumber: DHJPAR0006941; recordedBy: D.H. Janzen & W. Hallwachs, Manuel Rios; individualID: DHJPAR0006941; individualCount: 1; lifeStage: adult; preparations: pinned; otherCatalogNumbers: 06-SRNP-30178,ASTAV183-06; **Taxon:** scientificName: Cordyligaster capelli; phylum: Arthropoda; class: Insecta; order: Diptera; family: Tachinidae; genus: Cordyligaster; specificEpithet: capelli; scientificNameAuthorship: Fleming & Wood; **Location:** continent: Central America; country: Costa Rica; countryCode: CR; stateProvince: Guanacaste; county: Sector Pitilla; locality: Area de Conservacion Guanacaste; verbatimLocality: Pasmopa; verbatimElevation: 440; verbatimLatitude: 11.019; verbatimLongitude: -85.41; verbatimCoordinateSystem: Decimal; decimalLatitude: 11.019; decimalLongitude: -85.41; **Identification:** identifiedBy: AJ Fleming; dateIdentified: 2014; **Event:** samplingProtocol: Host Collection; verbatimEventDate: 29-Jan-2006; **Record Level:** language: en; institutionCode: CNC; collectionCode: Insects; basisOfRecord: Pinned Specimen
- **Type status:** Paratype. **Occurrence:** occurrenceDetails: http://janzen.sas.upenn.edu; catalogNumber: DHJPAR0006942; recordedBy: D.H. Janzen & W. Hallwachs, Manuel Rios; individualID: DHJPAR0006942; individualCount: 1; lifeStage: adult; preparations: pinned; otherCatalogNumbers: 06-SRNP-30172,ASTAV184-06; **Taxon:** scientificName: Cordyligaster capelli; phylum: Arthropoda; class: Insecta; order: Diptera; family: Tachinidae; genus: Cordyligaster; specificEpithet: capelli; scientificNameAuthorship: Fleming & Wood; **Location:** continent: Central America; country: Costa Rica; countryCode: CR; stateProvince: Guanacaste; county: Sector Pitilla; locality: Area de Conservacion Guanacaste; verbatimLocality: Pasmopa; verbatimElevation: 440; verbatimLatitude: 11.019; verbatimLongitude: -85.41; verbatimCoordinateSystem: Decimal; decimalLatitude: 11.019; decimalLongitude: -85.41; **Identification:** identifiedBy: AJ Fleming; dateIdentified: 2014; **Event:** samplingProtocol: Host Collection; verbatimEventDate: 29-Jan-2006; **Record Level:** language: en; institutionCode: CNC; collectionCode: Insects; basisOfRecord: Pinned Specimen
- **Type status:** Paratype. **Occurrence:** occurrenceDetails: http://janzen.sas.upenn.edu; catalogNumber: DHJPAR0006943; recordedBy: D.H. Janzen & W. Hallwachs, Manuel Rios; individualID: DHJPAR0006943; individualCount: 1; lifeStage: adult; preparations: pinned; otherCatalogNumbers: 06-SRNP-30171,ASTAV185-06; **Taxon:** scientificName: Cordyligaster capelli; phylum: Arthropoda; class: Insecta; order: Diptera; family: Tachinidae; genus: Cordyligaster; specificEpithet: capelli; scientificNameAuthorship: Fleming & Wood; **Location:** continent: Central America; country: Costa Rica; countryCode: CR; stateProvince: Guanacaste; county: Sector Pitilla; locality: Area de Conservacion Guanacaste; verbatimLocality: Pasmopa; verbatimElevation: 440; verbatimLatitude: 11.019; verbatimLongitude: -85.41; verbatimCoordinateSystem: Decimal; decimalLatitude: 11.019; decimalLongitude: -85.41; **Identification:** identifiedBy: AJ Fleming; dateIdentified: 2014; **Event:** samplingProtocol: Host Collection; verbatimEventDate: 30-Jan-2006; **Record Level:** language: en; institutionCode: CNC; collectionCode: Insects; basisOfRecord: Pinned Specimen
- **Type status:** Paratype. **Occurrence:** occurrenceDetails: http://janzen.sas.upenn.edu; catalogNumber: DHJPAR0006944; recordedBy: D.H. Janzen & W. Hallwachs, Petrona Rios; individualID: DHJPAR0006944; individualCount: 1; lifeStage: adult; preparations: pinned; otherCatalogNumbers: 06-SRNP-30863,ASTAV186-06; **Taxon:** scientificName: Cordyligaster capelli; phylum: Arthropoda; class: Insecta; order: Diptera; family: Tachinidae; genus: Cordyligaster; specificEpithet: capelli; scientificNameAuthorship: Fleming & Wood; **Location:** continent: Central America; country: Costa Rica; countryCode: CR; stateProvince: Guanacaste; county: Sector Pitilla; locality: Area de Conservacion Guanacaste; verbatimLocality: Pasmopa; verbatimElevation: 440; verbatimLatitude: 11.019; verbatimLongitude: -85.41; verbatimCoordinateSystem: Decimal; decimalLatitude: 11.019; decimalLongitude: -85.41; **Identification:** identifiedBy: AJ Fleming; dateIdentified: 2014; **Event:** samplingProtocol: Host Collection; verbatimEventDate: 28-Feb-2006; **Record Level:** language: en; institutionCode: CNC; collectionCode: Insects; basisOfRecord: Pinned Specimen
- **Type status:** Paratype. **Occurrence:** occurrenceDetails: http://janzen.sas.upenn.edu; catalogNumber: DHJPAR0006945; recordedBy: D.H. Janzen & W. Hallwachs, Manuel Rios; individualID: DHJPAR0006945; individualCount: 1; lifeStage: adult; preparations: pinned; otherCatalogNumbers: 06-SRNP-30168,ASTAV187-06; **Taxon:** scientificName: Cordyligaster capelli; phylum: Arthropoda; class: Insecta; order: Diptera; family: Tachinidae; genus: Cordyligaster; specificEpithet: capelli; scientificNameAuthorship: Fleming & Wood; **Location:** continent: Central America; country: Costa Rica; countryCode: CR; stateProvince: Guanacaste; county: Sector Pitilla; locality: Area de Conservacion Guanacaste; verbatimLocality: Pasmopa; verbatimElevation: 440; verbatimLatitude: 11.019; verbatimLongitude: -85.41; verbatimCoordinateSystem: Decimal; decimalLatitude: 11.019; decimalLongitude: -85.41; **Identification:** identifiedBy: AJ Fleming; dateIdentified: 2014; **Event:** samplingProtocol: Host Collection; verbatimEventDate: 30-Jan-2006; **Record Level:** language: en; institutionCode: CNC; collectionCode: Insects; basisOfRecord: Pinned Specimen
- **Type status:** Paratype. **Occurrence:** occurrenceDetails: http://janzen.sas.upenn.edu; catalogNumber: DHJPAR0006946; recordedBy: D.H. Janzen & W. Hallwachs, Manuel Rios; individualID: DHJPAR0006946; individualCount: 1; lifeStage: adult; preparations: pinned; otherCatalogNumbers: 06-SRNP-30017,ASTAV188-06; **Taxon:** scientificName: Cordyligaster capelli; phylum: Arthropoda; class: Insecta; order: Diptera; family: Tachinidae; genus: Cordyligaster; specificEpithet: capelli; scientificNameAuthorship: Fleming & Wood; **Location:** continent: Central America; country: Costa Rica; countryCode: CR; stateProvince: Guanacaste; county: Sector Pitilla; locality: Area de Conservacion Guanacaste; verbatimLocality: Pasmopa; verbatimElevation: 440; verbatimLatitude: 11.019; verbatimLongitude: -85.41; verbatimCoordinateSystem: Decimal; decimalLatitude: 11.019; decimalLongitude: -85.41; **Identification:** identifiedBy: AJ Fleming; dateIdentified: 2014; **Event:** samplingProtocol: Host Collection; verbatimEventDate: 28-Jan-2006; **Record Level:** language: en; institutionCode: CNC; collectionCode: Insects; basisOfRecord: Pinned Specimen
- **Type status:** Paratype. **Occurrence:** occurrenceDetails: http://janzen.sas.upenn.edu; catalogNumber: DHJPAR0055082; recordedBy: D.H. Janzen & W. Hallwachs, Keiner Aragon; individualID: DHJPAR0055082; individualCount: 1; lifeStage: adult; preparations: pinned; otherCatalogNumbers: 14-SRNP-45262,ASHYH1629-14; **Taxon:** scientificName: Cordyligaster capelli; phylum: Arthropoda; class: Insecta; order: Diptera; family: Tachinidae; genus: Cordyligaster; specificEpithet: capelli; scientificNameAuthorship: Fleming & Wood; **Location:** continent: Central America; country: Costa Rica; countryCode: CR; stateProvince: Alajuela; county: Sector Rincon Rain Forest; locality: Area de Conservacion Guanacaste; verbatimLocality: Palomo; verbatimElevation: 96; verbatimLatitude: 10.962; verbatimLongitude: -85.28; verbatimCoordinateSystem: Decimal; decimalLatitude: 10.962; decimalLongitude: -85.28; **Identification:** identifiedBy: AJ Fleming; dateIdentified: 2014; **Event:** samplingProtocol: Host Collection; verbatimEventDate: 18-Feb-2014; **Record Level:** language: en; institutionCode: CNC; collectionCode: Insects; basisOfRecord: Pinned Specimen

#### Description

Male (Fig. 1); Head: fronto orbital plate with silver tomentosity; parafacial silver; pedicel black; antenna black with plumose arista; trichiae at base, 6 times as long as base of arista is wide tapering to half that length before apex; eye bare; ocellar bristles parallel and proclinate approximately twice the length of the ocellar triangle; fronto-orbital plate narrowing at apex enclosing only the ocellar triangle; proclinate orbital bristles absent in male; palpus black. Thorax: at first glance appears glabrous black, but under certain angles of light a very light tomentum is often apparent however no vittae are visible. Three post-sutural supra alar bristles, (two strong anterior, and third one weak; second bristle strongest, 1.5X thickness of first post sutural supra alar bristles) (Fig. 3), apical scutellar bristles long, up to 3/4 length of subapical scutellars; subapical scutellar bristles parallel or divergent (forming a wide V); katepisternum bearing 2 bristles, very lightly tomentose (same as dorsum), lacking the tomentose bands apparent in *C. petiolata*. Wing: smoky yellow, dark amber towards base; vein R_1_  haired, vein R_4+5_ haired up to crossvein r-m (Fig. 4b); crossvein dm-cu straight not undulated; calypteres enlarged and translucent. Legs: black. Abdomen: petiolate with both discal bristles and median marginal bristles present on T1+2, T3, T4 and T5. Silver pollinosity on upper margins of abdominal segments T3, and T4. Very light tomentosity present on T5 but as in the case of the thorax, only visible under certain angles of light. Terminalia: surstylus equilaterally oblong shaped, apically bare, bearing many stout bristles posterodorsally, tip with strong inwardly apical curve when viewed dorsally. Cercus sharply pointed, ventral surface bare, separation between cerci narrow, up to 85% as long as surstylus. Dorsal surface of sternite 5 bearing two long bristles.

Female (Fig. 2); Head: fronto orbital plate with silver tomentosity except along facial ridge which appears red; pedicel black; antenna black with plumose  arista; trichiae at base, 6 times as long as base of arista is wide tapering to half that length before apex; eye bare; ocellar bristles parallel and proclinate approximately twice the length of the ocellar triangle; fronto-orbital plate narrowing at apex enclosing only the ocellar triangle; 2 proclinate orbital bristles present; palpus black, with distinctive gold tomentosity on tip. Thorax: at first glance appears glabrous black, however under certain angles of light a very light tomentum is apparent. Three post-sutural supra alar bristles, (two strong anterior, and third one weak); second bristle strongest, 1.5X thickness of first post-sutural supra alar; apical scutellar bristles long, equal in length of subapical scutellars; scutellar bristles divergent (forming a wide V); katepisternum bearing 2 bristles, very lightly tomentose (equal to dorsum), lacking the tomentose bands when viewed laterally that are apparent in *C. petiolata*. Wing: smoky yellow, dark amber towards base; vein R_1_ haired, vein R_4+5_ haired along ¾ of its length; crossvein dm-cu straight not undulated; calypteres enlarged and translucent. Legs: black. Abdomen: petiolate with both discal bristles and median marginal bristles present on T1+2, T3, T4 and T5. Silver pollinosity on upper margins of abdominal segments T3, and T4. Very light tomentosity present on T5 but as in the case of the thorax, this is only visible under certain angles of light.

#### Diagnosis

*Cordyligaster capellii* posesses exceptionally large calypteres, a trait also shared by *C. nyomala*, and *C. minuscula*. While *C. minuscula* (Wulp) can also sometimes have the variable character of 3 post-sutural supra alars, *C. capellii* is distinguished by the presence of 3 postsutural supra-alars (Fig. 3b), smoky yellow wings  (Fig. 4), and a black antennal pedicel and black palpi. *C. minuscula* possesses a orange brown pedicel, and reddish brown palpi. A very light tomentum covers much of the thorax as well as T5, but this trait is visible only under certain angles of light. *C. minuscula* also possesses only the posterior katepisternal bristle, while *C. capellii* has two. The holotype specimen has the DNA barcode given below:

ACTTTATATTTTATTTTTGGAGCATGAGCAGGTATATTAGGAACATCTTTAAGTATTTTAATTCGAACAGAATTAGGACATCCTGGTTCATTAATTGGAGATGATCAAATTTATAATGTTATTGTAACAGCTCATGCTTTTATTATAATTTTTTTTATAGTTATACCAATTATAATTGGAGGATTTGGAAATTGATTAGTTCCTTTAATATTAGGAGCACCAGATATAGCTTTCCCTCGAATAAATAATATAAGATTTTGACTTCTTCCTCCTTCTTTAATATTATTATTAGTTGGTAGAATAGTTGAAAATGGAGCTGGAACAGGATGAACTGTTTACCCTCCTTTATCTTCTAATATTGCTCATAGAGGATCTTCTGTAGATTTAACTATTTTTTCATTACATTTAGCAGGAATTTCTTCTATTATAGGAGCTGTAAATTTTATTACAACAGTAATTAATATACGAGCAACAGGAATTACATTTGATCGAATACCTTTATTTGTATGATCTGTAGCTATTACAGCTTTATTACTTTTATTATCATTACCTGTATTAGCCGGAGCTATTACTATATTATTAACAGATCGAAATATAAATACTTCATTTTTTGATCCTGCAGGAGGAGGAGATCCTATTTTATATCAACACTTATTT.

Genetic comparison to the type specimens of previously know species was outside the scope of this paper, however the authors have selected to give the barcode data here as a diagnostic character such that it is readily available for future works which may undertake the barcoding of those previously described types.

#### Etymology

This species is named to honor Sr. Luciano Capelli of San Jose, Costa Rica in recognition and appreciation of his enthusiastic and superb photography of all aspects of ACG specifically, and Costa Rica’s conserved wildlands more broadly, and for allowing ACG and Costa Rican conservation in general to freely use these photographs to explain conserved wildlands to the public.

#### Distribution

Costa Rica, ACG, Prov. Guanacaste, rain forest, 390 – 440m  m elevation.

#### Ecology

**Hosts:** Crambidae, *Syngamia florella* (Stoll, 1781).   While more than 500 species of Crambidae have been reared from more than 65,000 leaf-rolling crambid caterpillars in ACG dry forest, rain forest and cloud forest (and intergrades among them), generating 3,000+ tachinid rearings, *Cordyligaster capellii* has been reared just 10 times and always from the leaf roller *Syngamia florella* feeding on *Spermacoce exilis* (L.O. Williams) (Rubiaceae) herbs in the dry-rain forest ecotone on the northern intermediate elevation slopes of Volcan Orosi and Cerro Orosilito, Sector Del Oro and Sector Pitilla, of ACG.  These ten rearings were spread among 144 *S. florella* wild-caught caterpillars. It is likely to be the only species of host for this fly in ACG.

### Cordyligaster fuscipennis

(Macquart, 1851)

#### Materials

- **Type status:** Other material. **Occurrence:** occurrenceDetails: http://janzen.sas.upenn.edu; catalogNumber: DHJPAR0006720; recordedBy: D.H. Janzen & W. Hallwachs; individualID: DHJPAR0006720; individualCount: 1; lifeStage: adult; preparations: pinned; otherCatalogNumbers: ASTA898-06,06-SRNP-40070; **Taxon:** scientificName: Cordyligaster petiolata; phylum: Arthropoda; class: Insecta; order: Diptera; family: Tachinidae; genus: Cordyligaster; specificEpithet: fuscipennis; scientificNameAuthorship: (Macquart, 1851); **Location:** continent: Central America; country: Costa Rica; countryCode: CR; stateProvince: Alajuela; county: Sector Rincon Rain Forest; locality: Area de Conservacion Guanacaste; verbatimLocality: Sendero Juntas; verbatimElevation: 400; verbatimLatitude: 10.907; verbatimLongitude: -85.288; verbatimCoordinateSystem: Decimal; decimalLatitude: 10.907; decimalLongitude: -85.288; **Identification:** identifiedBy: AJ Fleming; dateIdentified: 2014; **Event:** samplingProtocol: Host Collection; verbatimEventDate: 01-Feb-2006; **Record Level:** language: en; institutionCode: CNC; collectionCode: Insects; basisOfRecord: Pinned Specimen
- **Type status:** Other material. **Occurrence:** occurrenceDetails: http://janzen.sas.upenn.edu; catalogNumber: DHJPAR0006723; recordedBy: D.H. Janzen & W. Hallwachs; individualID: DHJPAR0006723; individualCount: 1; lifeStage: adult; preparations: pinned; otherCatalogNumbers: ASTA901-06,05-SRNP-43874; **Taxon:** scientificName: Cordyligaster petiolata; phylum: Arthropoda; class: Insecta; order: Diptera; family: Tachinidae; genus: Cordyligaster; specificEpithet: fuscipennis; scientificNameAuthorship: (Macquart, 1851); **Location:** continent: Central America; country: Costa Rica; countryCode: CR; stateProvince: Alajuela; county: Sector Rincon Rain Forest; locality: Area de Conservacion Guanacaste; verbatimLocality: Rio Francia Arriba; verbatimElevation: 400; verbatimLatitude: 10.897; verbatimLongitude: -85.29; verbatimCoordinateSystem: Decimal; decimalLatitude: 10.897; decimalLongitude: -85.29; **Identification:** identifiedBy: AJ Fleming; dateIdentified: 2014; **Event:** samplingProtocol: Host Collection; verbatimEventDate: 18-Jan-2006; **Record Level:** language: en; institutionCode: CNC; collectionCode: Insects; basisOfRecord: Pinned Specimen
- **Type status:** Other material. **Occurrence:** occurrenceDetails: http://janzen.sas.upenn.edu; catalogNumber: DHJPAR0006724; recordedBy: D.H. Janzen & W. Hallwachs; individualID: DHJPAR0006724; individualCount: 1; lifeStage: adult; preparations: pinned; otherCatalogNumbers: ASTA902-06,05-SRNP-43875; **Taxon:** scientificName: Cordyligaster petiolata; phylum: Arthropoda; class: Insecta; order: Diptera; family: Tachinidae; genus: Cordyligaster; specificEpithet: fuscipennis; scientificNameAuthorship: (Macquart, 1851); **Location:** continent: Central America; country: Costa Rica; countryCode: CR; stateProvince: Alajuela; county: Sector Rincon Rain Forest; locality: Area de Conservacion Guanacaste; verbatimLocality: Rio Francia Arriba; verbatimElevation: 400; verbatimLatitude: 10.897; verbatimLongitude: -85.29; verbatimCoordinateSystem: Decimal; decimalLatitude: 10.897; decimalLongitude: -85.29; **Identification:** identifiedBy: AJ Fleming; dateIdentified: 2014; **Event:** samplingProtocol: Host Collection; verbatimEventDate: 21-Jan-2006; **Record Level:** language: en; institutionCode: CNC; collectionCode: Insects; basisOfRecord: Pinned Specimen
- **Type status:** Other material. **Occurrence:** occurrenceDetails: http://janzen.sas.upenn.edu; catalogNumber: DHJPAR0006935; recordedBy: D.H. Janzen & W. Hallwachs; individualID: DHJPAR0006935; individualCount: 1; lifeStage: adult; preparations: pinned; otherCatalogNumbers: ASTAV177-06,06-SRNP-40889; **Taxon:** scientificName: Cordyligaster petiolata; phylum: Arthropoda; class: Insecta; order: Diptera; family: Tachinidae; genus: Cordyligaster; specificEpithet: fuscipennis; scientificNameAuthorship: (Macquart, 1851); **Location:** continent: Central America; country: Costa Rica; countryCode: CR; stateProvince: Alajuela; county: Sector Rincon Rain Forest; locality: Area de Conservacion Guanacaste; verbatimLocality: Sendero Juntas; verbatimElevation: 400; verbatimLatitude: 10.907; verbatimLongitude: -85.288; verbatimCoordinateSystem: Decimal; decimalLatitude: 10.907; decimalLongitude: -85.288; **Identification:** identifiedBy: AJ Fleming; dateIdentified: 2014; **Event:** samplingProtocol: Host Collection; verbatimEventDate: 29-Mar-2006; **Record Level:** language: en; institutionCode: CNC; collectionCode: Insects; basisOfRecord: Pinned Specimen
- **Type status:** Other material. **Occurrence:** occurrenceDetails: http://janzen.sas.upenn.edu; catalogNumber: DHJPAR0006936; recordedBy: D.H. Janzen & W. Hallwachs; individualID: DHJPAR0006936; individualCount: 1; lifeStage: adult; preparations: pinned; otherCatalogNumbers: ASTAV178-06,06-SRNP-40664; **Taxon:** scientificName: Cordyligaster petiolata; phylum: Arthropoda; class: Insecta; order: Diptera; family: Tachinidae; genus: Cordyligaster; specificEpithet: fuscipennis; scientificNameAuthorship: (Macquart, 1851); **Location:** continent: Central America; country: Costa Rica; countryCode: CR; stateProvince: Alajuela; county: Sector Rincon Rain Forest; locality: Area de Conservacion Guanacaste; verbatimLocality: Sendero Juntas; verbatimElevation: 400; verbatimLatitude: 10.907; verbatimLongitude: -85.288; verbatimCoordinateSystem: Decimal; decimalLatitude: 10.907; decimalLongitude: -85.288; **Identification:** identifiedBy: AJ Fleming; dateIdentified: 2014; **Event:** samplingProtocol: Host Collection; verbatimEventDate: 13-Mar-2006; **Record Level:** language: en; institutionCode: CNC; collectionCode: Insects; basisOfRecord: Pinned Specimen
- **Type status:** Other material. **Occurrence:** occurrenceDetails: http://janzen.sas.upenn.edu; catalogNumber: DHJPAR0006937; recordedBy: D.H. Janzen & W. Hallwachs; individualID: DHJPAR0006937; individualCount: 1; lifeStage: adult; preparations: pinned; otherCatalogNumbers: ASTAV179-06,06-SRNP-40669; **Taxon:** scientificName: Cordyligaster petiolata; phylum: Arthropoda; class: Insecta; order: Diptera; family: Tachinidae; genus: Cordyligaster; specificEpithet: fuscipennis; scientificNameAuthorship: (Macquart, 1851); **Location:** continent: Central America; country: Costa Rica; countryCode: CR; stateProvince: Alajuela; county: Sector Rincon Rain Forest; locality: Area de Conservacion Guanacaste; verbatimLocality: Sendero Juntas; verbatimElevation: 400; verbatimLatitude: 10.907; verbatimLongitude: -85.288; verbatimCoordinateSystem: Decimal; decimalLatitude: 10.907; decimalLongitude: -85.288; **Identification:** identifiedBy: AJ Fleming; dateIdentified: 2014; **Event:** samplingProtocol: Host Collection; verbatimEventDate: 17-Mar-2006; **Record Level:** language: en; institutionCode: CNC; collectionCode: Insects; basisOfRecord: Pinned Specimen
- **Type status:** Other material. **Occurrence:** occurrenceDetails: http://janzen.sas.upenn.edu; catalogNumber: DHJPAR0010199; recordedBy: D.H. Janzen & W. Hallwachs; individualID: DHJPAR0010199; individualCount: 1; lifeStage: adult; preparations: pinned; otherCatalogNumbers: ASTAV725-06,06-SRNP-41615; **Taxon:** scientificName: Cordyligaster petiolata; phylum: Arthropoda; class: Insecta; order: Diptera; family: Tachinidae; genus: Cordyligaster; specificEpithet: fuscipennis; scientificNameAuthorship: (Macquart, 1851); **Location:** continent: Central America; country: Costa Rica; countryCode: CR; stateProvince: Alajuela; county: Sector Rincon Rain Forest; locality: Area de Conservacion Guanacaste; verbatimLocality: Rio Francia Arriba; verbatimElevation: 400; verbatimLatitude: 10.897; verbatimLongitude: -85.29; verbatimCoordinateSystem: Decimal; decimalLatitude: 10.897; decimalLongitude: -85.29; **Identification:** identifiedBy: AJ Fleming; dateIdentified: 2014; **Event:** samplingProtocol: Host Collection; verbatimEventDate: 23-May-2006; **Record Level:** language: en; institutionCode: CNC; collectionCode: Insects; basisOfRecord: Pinned Specimen
- **Type status:** Other material. **Occurrence:** occurrenceDetails: http://janzen.sas.upenn.edu; catalogNumber: DHJPAR0010200; recordedBy: D.H. Janzen & W. Hallwachs; individualID: DHJPAR0010200; individualCount: 1; lifeStage: adult; preparations: pinned; otherCatalogNumbers: ASTAV726-06,06-SRNP-41577; **Taxon:** scientificName: Cordyligaster petiolata; phylum: Arthropoda; class: Insecta; order: Diptera; family: Tachinidae; genus: Cordyligaster; specificEpithet: fuscipennis; scientificNameAuthorship: (Macquart, 1851); **Location:** continent: Central America; country: Costa Rica; countryCode: CR; stateProvince: Alajuela; county: Sector Rincon Rain Forest; locality: Area de Conservacion Guanacaste; verbatimLocality: Rio Francia Arriba; verbatimElevation: 400; verbatimLatitude: 10.897; verbatimLongitude: -85.29; verbatimCoordinateSystem: Decimal; decimalLatitude: 10.897; decimalLongitude: -85.29; **Identification:** identifiedBy: AJ Fleming; dateIdentified: 2014; **Event:** samplingProtocol: Host Collection; verbatimEventDate: 22-May-2006; **Record Level:** language: en; institutionCode: CNC; collectionCode: Insects; basisOfRecord: Pinned Specimen
- **Type status:** Other material. **Occurrence:** occurrenceDetails: http://janzen.sas.upenn.edu; catalogNumber: DHJPAR0010201; recordedBy: D.H. Janzen & W. Hallwachs; individualID: DHJPAR0010201; individualCount: 1; lifeStage: adult; preparations: pinned; otherCatalogNumbers: ASTAV727-06,06-SRNP-41595; **Taxon:** scientificName: Cordyligaster petiolata; phylum: Arthropoda; class: Insecta; order: Diptera; family: Tachinidae; genus: Cordyligaster; specificEpithet: fuscipennis; scientificNameAuthorship: (Macquart, 1851); **Location:** continent: Central America; country: Costa Rica; countryCode: CR; stateProvince: Alajuela; county: Sector Rincon Rain Forest; locality: Area de Conservacion Guanacaste; verbatimLocality: Rio Francia Arriba; verbatimElevation: 400; verbatimLatitude: 10.897; verbatimLongitude: -85.29; verbatimCoordinateSystem: Decimal; decimalLatitude: 10.897; decimalLongitude: -85.29; **Identification:** identifiedBy: AJ Fleming; dateIdentified: 2014; **Event:** samplingProtocol: Host Collection; verbatimEventDate: 23-May-2006; **Record Level:** language: en; institutionCode: CNC; collectionCode: Insects; basisOfRecord: Pinned Specimen
- **Type status:** Other material. **Occurrence:** occurrenceDetails: http://janzen.sas.upenn.edu; catalogNumber: DHJPAR0010202; recordedBy: D.H. Janzen & W. Hallwachs; individualID: DHJPAR0010202; individualCount: 1; lifeStage: adult; preparations: pinned; otherCatalogNumbers: ASTAV728-06,06-SRNP-41589; **Taxon:** scientificName: Cordyligaster petiolata; phylum: Arthropoda; class: Insecta; order: Diptera; family: Tachinidae; genus: Cordyligaster; specificEpithet: fuscipennis; scientificNameAuthorship: (Macquart, 1851); **Location:** continent: Central America; country: Costa Rica; countryCode: CR; stateProvince: Alajuela; county: Sector Rincon Rain Forest; locality: Area de Conservacion Guanacaste; verbatimLocality: Rio Francia Arriba; verbatimElevation: 400; verbatimLatitude: 10.897; verbatimLongitude: -85.29; verbatimCoordinateSystem: Decimal; decimalLatitude: 10.897; decimalLongitude: -85.29; **Identification:** identifiedBy: AJ Fleming; dateIdentified: 2014; **Event:** samplingProtocol: Host Collection; verbatimEventDate: 21-May-2006; **Record Level:** language: en; institutionCode: CNC; collectionCode: Insects; basisOfRecord: Pinned Specimen
- **Type status:** Other material. **Occurrence:** occurrenceDetails: http://janzen.sas.upenn.edu; catalogNumber: DHJPAR0010205; recordedBy: D.H. Janzen & W. Hallwachs; individualID: DHJPAR0010205; individualCount: 1; lifeStage: adult; preparations: pinned; otherCatalogNumbers: ASTAV731-06,06-SRNP-41628; **Taxon:** scientificName: Cordyligaster petiolata; phylum: Arthropoda; class: Insecta; order: Diptera; family: Tachinidae; genus: Cordyligaster; specificEpithet: fuscipennis; scientificNameAuthorship: (Macquart, 1851); **Location:** continent: Central America; country: Costa Rica; countryCode: CR; stateProvince: Alajuela; county: Sector Rincon Rain Forest; locality: Area de Conservacion Guanacaste; verbatimLocality: Rio Francia Arriba; verbatimElevation: 400; verbatimLatitude: 10.897; verbatimLongitude: -85.29; verbatimCoordinateSystem: Decimal; decimalLatitude: 10.897; decimalLongitude: -85.29; **Identification:** identifiedBy: AJ Fleming; dateIdentified: 2014; **Event:** samplingProtocol: Host Collection; verbatimEventDate: 24-May-2006; **Record Level:** language: en; institutionCode: CNC; collectionCode: Insects; basisOfRecord: Pinned Specimen
- **Type status:** Other material. **Occurrence:** occurrenceDetails: http://janzen.sas.upenn.edu; catalogNumber: DHJPAR0010206; recordedBy: D.H. Janzen & W. Hallwachs; individualID: DHJPAR0010206; individualCount: 1; lifeStage: adult; preparations: pinned; otherCatalogNumbers: ASTAV732-06,06-SRNP-41578; **Taxon:** scientificName: Cordyligaster petiolata; phylum: Arthropoda; class: Insecta; order: Diptera; family: Tachinidae; genus: Cordyligaster; specificEpithet: fuscipennis; scientificNameAuthorship: (Macquart, 1851); **Location:** continent: Central America; country: Costa Rica; countryCode: CR; stateProvince: Alajuela; county: Sector Rincon Rain Forest; locality: Area de Conservacion Guanacaste; verbatimLocality: Rio Francia Arriba; verbatimElevation: 400; verbatimLatitude: 10.897; verbatimLongitude: -85.29; verbatimCoordinateSystem: Decimal; decimalLatitude: 10.897; decimalLongitude: -85.29; **Identification:** identifiedBy: AJ Fleming; dateIdentified: 2014; **Event:** samplingProtocol: Host Collection; verbatimEventDate: 25-May-2006; **Record Level:** language: en; institutionCode: CNC; collectionCode: Insects; basisOfRecord: Pinned Specimen
- **Type status:** Other material. **Occurrence:** occurrenceDetails: http://janzen.sas.upenn.edu; catalogNumber: DHJPAR0010207; recordedBy: D.H. Janzen & W. Hallwachs; individualID: DHJPAR0010207; individualCount: 1; lifeStage: adult; preparations: pinned; otherCatalogNumbers: ASTAV733-06,06-SRNP-41600; **Taxon:** scientificName: Cordyligaster petiolata; phylum: Arthropoda; class: Insecta; order: Diptera; family: Tachinidae; genus: Cordyligaster; specificEpithet: fuscipennis; scientificNameAuthorship: (Macquart, 1851); **Location:** continent: Central America; country: Costa Rica; countryCode: CR; stateProvince: Alajuela; county: Sector Rincon Rain Forest; locality: Area de Conservacion Guanacaste; verbatimLocality: Rio Francia Arriba; verbatimElevation: 400; verbatimLatitude: 10.897; verbatimLongitude: -85.29; verbatimCoordinateSystem: Decimal; decimalLatitude: 10.897; decimalLongitude: -85.29; **Identification:** identifiedBy: AJ Fleming; dateIdentified: 2014; **Event:** samplingProtocol: Host Collection; verbatimEventDate: 24-May-2006; **Record Level:** language: en; institutionCode: CNC; collectionCode: Insects; basisOfRecord: Pinned Specimen
- **Type status:** Other material. **Occurrence:** occurrenceDetails: http://janzen.sas.upenn.edu; catalogNumber: DHJPAR0010208; recordedBy: D.H. Janzen & W. Hallwachs; individualID: DHJPAR0010208; individualCount: 1; lifeStage: adult; preparations: pinned; otherCatalogNumbers: ASTAV734-06,06-SRNP-41613; **Taxon:** scientificName: Cordyligaster petiolata; phylum: Arthropoda; class: Insecta; order: Diptera; family: Tachinidae; genus: Cordyligaster; specificEpithet: fuscipennis; scientificNameAuthorship: (Macquart, 1851); **Location:** continent: Central America; country: Costa Rica; countryCode: CR; stateProvince: Alajuela; county: Sector Rincon Rain Forest; locality: Area de Conservacion Guanacaste; verbatimLocality: Rio Francia Arriba; verbatimElevation: 400; verbatimLatitude: 10.897; verbatimLongitude: -85.29; verbatimCoordinateSystem: Decimal; decimalLatitude: 10.897; decimalLongitude: -85.29; **Identification:** identifiedBy: AJ Fleming; dateIdentified: 2014; **Event:** samplingProtocol: Host Collection; verbatimEventDate: 24-May-2006; **Record Level:** language: en; institutionCode: CNC; collectionCode: Insects; basisOfRecord: Pinned Specimen
- **Type status:** Other material. **Occurrence:** occurrenceDetails: http://janzen.sas.upenn.edu; catalogNumber: DHJPAR0010209; recordedBy: D.H. Janzen & W. Hallwachs; individualID: DHJPAR0010209; individualCount: 1; lifeStage: adult; preparations: pinned; otherCatalogNumbers: ASTAV735-06,06-SRNP-41579; **Taxon:** scientificName: Cordyligaster petiolata; phylum: Arthropoda; class: Insecta; order: Diptera; family: Tachinidae; genus: Cordyligaster; specificEpithet: fuscipennis; scientificNameAuthorship: (Macquart, 1851); **Location:** continent: Central America; country: Costa Rica; countryCode: CR; stateProvince: Alajuela; county: Sector Rincon Rain Forest; locality: Area de Conservacion Guanacaste; verbatimLocality: Rio Francia Arriba; verbatimElevation: 400; verbatimLatitude: 10.897; verbatimLongitude: -85.29; verbatimCoordinateSystem: Decimal; decimalLatitude: 10.897; decimalLongitude: -85.29; **Identification:** identifiedBy: AJ Fleming; dateIdentified: 2014; **Event:** samplingProtocol: Host Collection; verbatimEventDate: 24-May-2006; **Record Level:** language: en; institutionCode: CNC; collectionCode: Insects; basisOfRecord: Pinned Specimen
- **Type status:** Other material. **Occurrence:** occurrenceDetails: http://janzen.sas.upenn.edu; catalogNumber: DHJPAR0010210; recordedBy: D.H. Janzen & W. Hallwachs; individualID: DHJPAR0010210; individualCount: 1; lifeStage: adult; preparations: pinned; otherCatalogNumbers: ASTAV736-06,06-SRNP-41616; **Taxon:** scientificName: Cordyligaster petiolata; phylum: Arthropoda; class: Insecta; order: Diptera; family: Tachinidae; genus: Cordyligaster; specificEpithet: fuscipennis; scientificNameAuthorship: (Macquart, 1851); **Location:** continent: Central America; country: Costa Rica; countryCode: CR; stateProvince: Alajuela; county: Sector Rincon Rain Forest; locality: Area de Conservacion Guanacaste; verbatimLocality: Rio Francia Arriba; verbatimElevation: 400; verbatimLatitude: 10.897; verbatimLongitude: -85.29; verbatimCoordinateSystem: Decimal; decimalLatitude: 10.897; decimalLongitude: -85.29; **Identification:** identifiedBy: AJ Fleming; dateIdentified: 2014; **Event:** samplingProtocol: Host Collection; verbatimEventDate: 24-May-2006; **Record Level:** language: en; institutionCode: CNC; collectionCode: Insects; basisOfRecord: Pinned Specimen
- **Type status:** Other material. **Occurrence:** occurrenceDetails: http://janzen.sas.upenn.edu; catalogNumber: DHJPAR0010212; recordedBy: D.H. Janzen & W. Hallwachs; individualID: DHJPAR0010212; individualCount: 1; lifeStage: adult; preparations: pinned; otherCatalogNumbers: ASTAV738-06,06-SRNP-41582; **Taxon:** scientificName: Cordyligaster petiolata; phylum: Arthropoda; class: Insecta; order: Diptera; family: Tachinidae; genus: Cordyligaster; specificEpithet: fuscipennis; scientificNameAuthorship: (Macquart, 1851); **Location:** continent: Central America; country: Costa Rica; countryCode: CR; stateProvince: Alajuela; county: Sector Rincon Rain Forest; locality: Area de Conservacion Guanacaste; verbatimLocality: Rio Francia Arriba; verbatimElevation: 400; verbatimLatitude: 10.897; verbatimLongitude: -85.29; verbatimCoordinateSystem: Decimal; decimalLatitude: 10.897; decimalLongitude: -85.29; **Identification:** identifiedBy: AJ Fleming; dateIdentified: 2014; **Event:** samplingProtocol: Host Collection; verbatimEventDate: 27-May-2006; **Record Level:** language: en; institutionCode: CNC; collectionCode: Insects; basisOfRecord: Pinned Specimen
- **Type status:** Other material. **Occurrence:** occurrenceDetails: http://janzen.sas.upenn.edu; catalogNumber: DHJPAR0010213; recordedBy: D.H. Janzen & W. Hallwachs; individualID: DHJPAR0010213; individualCount: 1; lifeStage: adult; preparations: pinned; otherCatalogNumbers: ASTAV739-06,06-SRNP-41586; **Taxon:** scientificName: Cordyligaster petiolata; phylum: Arthropoda; class: Insecta; order: Diptera; family: Tachinidae; genus: Cordyligaster; specificEpithet: fuscipennis; scientificNameAuthorship: (Macquart, 1851); **Location:** continent: Central America; country: Costa Rica; countryCode: CR; stateProvince: Alajuela; county: Sector Rincon Rain Forest; locality: Area de Conservacion Guanacaste; verbatimLocality: Rio Francia Arriba; verbatimElevation: 400; verbatimLatitude: 10.897; verbatimLongitude: -85.29; verbatimCoordinateSystem: Decimal; decimalLatitude: 10.897; decimalLongitude: -85.29; **Identification:** identifiedBy: AJ Fleming; dateIdentified: 2014; **Event:** samplingProtocol: Host Collection; verbatimEventDate: 26-May-2006; **Record Level:** language: en; institutionCode: CNC; collectionCode: Insects; basisOfRecord: Pinned Specimen
- **Type status:** Other material. **Occurrence:** occurrenceDetails: http://janzen.sas.upenn.edu; catalogNumber: DHJPAR0019634; recordedBy: D.H. Janzen & W. Hallwachs; individualID: DHJPAR0019634; individualCount: 1; lifeStage: adult; preparations: pinned; otherCatalogNumbers: ASTAB182-07,07-SRNP-40768; **Taxon:** scientificName: Cordyligaster petiolata; phylum: Arthropoda; class: Insecta; order: Diptera; family: Tachinidae; genus: Cordyligaster; specificEpithet: fuscipennis; scientificNameAuthorship: (Macquart, 1851); **Location:** continent: Central America; country: Costa Rica; countryCode: CR; stateProvince: Alajuela; county: Sector Rincon Rain Forest; locality: Area de Conservacion Guanacaste; verbatimLocality: Sendero Juntas; verbatimElevation: 400; verbatimLatitude: 10.907; verbatimLongitude: -85.288; verbatimCoordinateSystem: Decimal; decimalLatitude: 10.907; decimalLongitude: -85.288; **Identification:** identifiedBy: AJ Fleming; dateIdentified: 2014; **Event:** samplingProtocol: Host Collection; verbatimEventDate: 11-Apr-2007; **Record Level:** language: en; institutionCode: CNC; collectionCode: Insects; basisOfRecord: Pinned Specimen
- **Type status:** Other material. **Occurrence:** occurrenceDetails: http://janzen.sas.upenn.edu; catalogNumber: DHJPAR0030036; recordedBy: D.H. Janzen & W. Hallwachs; individualID: DHJPAR0030036; individualCount: 1; lifeStage: adult; preparations: pinned; otherCatalogNumbers: ASHYB780-09,09-SRNP-243; **Taxon:** scientificName: Cordyligaster petiolata; phylum: Arthropoda; class: Insecta; order: Diptera; family: Tachinidae; genus: Cordyligaster; specificEpithet: fuscipennis; scientificNameAuthorship: (Macquart, 1851); **Location:** continent: Central America; country: Costa Rica; countryCode: CR; stateProvince: Alajuela; county: Sector San Cristobal; locality: Area de Conservacion Guanacaste; verbatimLocality: Sendero Huerta; verbatimElevation: 527; verbatimLatitude: 10.931; verbatimLongitude: -85.372; verbatimCoordinateSystem: Decimal; decimalLatitude: 10.931; decimalLongitude: -85.372; **Identification:** identifiedBy: AJ Fleming; dateIdentified: 2014; **Event:** samplingProtocol: Host Collection; verbatimEventDate: 12-Feb-2009; **Record Level:** language: en; institutionCode: CNC; collectionCode: Insects; basisOfRecord: Pinned Specimen
- **Type status:** Other material. **Occurrence:** occurrenceDetails: http://janzen.sas.upenn.edu; catalogNumber: DHJPAR0030037; recordedBy: D.H. Janzen & W. Hallwachs; individualID: DHJPAR0030037; individualCount: 1; lifeStage: adult; preparations: pinned; otherCatalogNumbers: ASHYB781-09,09-SRNP-246; **Taxon:** scientificName: Cordyligaster petiolata; phylum: Arthropoda; class: Insecta; order: Diptera; family: Tachinidae; genus: Cordyligaster; specificEpithet: fuscipennis; scientificNameAuthorship: (Macquart, 1851); **Location:** continent: Central America; country: Costa Rica; countryCode: CR; stateProvince: Alajuela; county: Sector San Cristobal; locality: Area de Conservacion Guanacaste; verbatimLocality: Sendero Huerta; verbatimElevation: 527; verbatimLatitude: 10.931; verbatimLongitude: -85.372; verbatimCoordinateSystem: Decimal; decimalLatitude: 10.931; decimalLongitude: -85.372; **Identification:** identifiedBy: AJ Fleming; dateIdentified: 2014; **Event:** samplingProtocol: Host Collection; verbatimEventDate: 14-Feb-2009; **Record Level:** language: en; institutionCode: CNC; collectionCode: Insects; basisOfRecord: Pinned Specimen
- **Type status:** Other material. **Occurrence:** occurrenceDetails: http://janzen.sas.upenn.edu; catalogNumber: DHJPAR0030038; recordedBy: D.H. Janzen & W. Hallwachs; individualID: DHJPAR0030038; individualCount: 1; lifeStage: adult; preparations: pinned; otherCatalogNumbers: ASHYB782-09,09-SRNP-250; **Taxon:** scientificName: Cordyligaster petiolata; phylum: Arthropoda; class: Insecta; order: Diptera; family: Tachinidae; genus: Cordyligaster; specificEpithet: fuscipennis; scientificNameAuthorship: (Macquart, 1851); **Location:** continent: Central America; country: Costa Rica; countryCode: CR; stateProvince: Alajuela; county: Sector San Cristobal; locality: Area de Conservacion Guanacaste; verbatimLocality: Sendero Huerta; verbatimElevation: 527; verbatimLatitude: 10.931; verbatimLongitude: -85.372; verbatimCoordinateSystem: Decimal; decimalLatitude: 10.931; decimalLongitude: -85.372; **Identification:** identifiedBy: AJ Fleming; dateIdentified: 2014; **Event:** samplingProtocol: Host Collection; verbatimEventDate: 16-Feb-2009; **Record Level:** language: en; institutionCode: CNC; collectionCode: Insects; basisOfRecord: Pinned Specimen
- **Type status:** Other material. **Occurrence:** occurrenceDetails: http://janzen.sas.upenn.edu; catalogNumber: DHJPAR0030039; recordedBy: D.H. Janzen & W. Hallwachs; individualID: DHJPAR0030039; individualCount: 1; lifeStage: adult; preparations: pinned; otherCatalogNumbers: ASHYB783-09,09-SRNP-254; **Taxon:** scientificName: Cordyligaster petiolata; phylum: Arthropoda; class: Insecta; order: Diptera; family: Tachinidae; genus: Cordyligaster; specificEpithet: fuscipennis; scientificNameAuthorship: (Macquart, 1851); **Location:** continent: Central America; country: Costa Rica; countryCode: CR; stateProvince: Alajuela; county: Sector San Cristobal; locality: Area de Conservacion Guanacaste; verbatimLocality: Sendero Huerta; verbatimElevation: 527; verbatimLatitude: 10.931; verbatimLongitude: -85.372; verbatimCoordinateSystem: Decimal; decimalLatitude: 10.931; decimalLongitude: -85.372; **Identification:** identifiedBy: AJ Fleming; dateIdentified: 2014; **Event:** samplingProtocol: Host Collection; verbatimEventDate: 06-Feb-2009; **Record Level:** language: en; institutionCode: CNC; collectionCode: Insects; basisOfRecord: Pinned Specimen
- **Type status:** Other material. **Occurrence:** occurrenceDetails: http://janzen.sas.upenn.edu; catalogNumber: DHJPAR0030462; recordedBy: D.H. Janzen & W. Hallwachs; individualID: DHJPAR0030462; individualCount: 1; lifeStage: adult; preparations: pinned; otherCatalogNumbers: ASHYB1205-09,09-SRNP-242; **Taxon:** scientificName: Cordyligaster petiolata; phylum: Arthropoda; class: Insecta; order: Diptera; family: Tachinidae; genus: Cordyligaster; specificEpithet: fuscipennis; scientificNameAuthorship: (Macquart, 1851); **Location:** continent: Central America; country: Costa Rica; countryCode: CR; stateProvince: Alajuela; county: Sector San Cristobal; locality: Area de Conservacion Guanacaste; verbatimLocality: Sendero Huerta; verbatimElevation: 527; verbatimLatitude: 10.931; verbatimLongitude: -85.372; verbatimCoordinateSystem: Decimal; decimalLatitude: 10.931; decimalLongitude: -85.372; **Identification:** identifiedBy: AJ Fleming; dateIdentified: 2014; **Event:** samplingProtocol: Host Collection; verbatimEventDate: 12-Feb-2009; **Record Level:** language: en; institutionCode: CNC; collectionCode: Insects; basisOfRecord: Pinned Specimen
- **Type status:** Other material. **Occurrence:** occurrenceDetails: http://janzen.sas.upenn.edu; catalogNumber: DHJPAR0030463; recordedBy: D.H. Janzen & W. Hallwachs; individualID: DHJPAR0030463; individualCount: 1; lifeStage: adult; preparations: pinned; otherCatalogNumbers: ASHYB1206-09,09-SRNP-256; **Taxon:** scientificName: Cordyligaster petiolata; phylum: Arthropoda; class: Insecta; order: Diptera; family: Tachinidae; genus: Cordyligaster; specificEpithet: fuscipennis; scientificNameAuthorship: (Macquart, 1851); **Location:** continent: Central America; country: Costa Rica; countryCode: CR; stateProvince: Alajuela; county: Sector San Cristobal; locality: Area de Conservacion Guanacaste; verbatimLocality: Sendero Huerta; verbatimElevation: 527; verbatimLatitude: 10.931; verbatimLongitude: -85.372; verbatimCoordinateSystem: Decimal; decimalLatitude: 10.931; decimalLongitude: -85.372; **Identification:** identifiedBy: AJ Fleming; dateIdentified: 2014; **Event:** samplingProtocol: Host Collection; verbatimEventDate: 04-Feb-2009; **Record Level:** language: en; institutionCode: CNC; collectionCode: Insects; basisOfRecord: Pinned Specimen
- **Type status:** Other material. **Occurrence:** occurrenceDetails: http://janzen.sas.upenn.edu; catalogNumber: DHJPAR0037670; recordedBy: D.H. Janzen & W. Hallwachs; individualID: DHJPAR0037670; individualCount: 1; lifeStage: adult; preparations: pinned; otherCatalogNumbers: ASHYC4415-10,10-SRNP-40318; **Taxon:** scientificName: Cordyligaster petiolata; phylum: Arthropoda; class: Insecta; order: Diptera; family: Tachinidae; genus: Cordyligaster; specificEpithet: fuscipennis; scientificNameAuthorship: (Macquart, 1851); **Location:** continent: Central America; country: Costa Rica; countryCode: CR; stateProvince: Alajuela; county: Sector Rincon Rain Forest; locality: Area de Conservacion Guanacaste; verbatimLocality: Sendero Juntas; verbatimElevation: 400; verbatimLatitude: 10.907; verbatimLongitude: -85.288; verbatimCoordinateSystem: Decimal; decimalLatitude: 10.907; decimalLongitude: -85.288; **Identification:** identifiedBy: AJ Fleming; dateIdentified: 2014; **Event:** samplingProtocol: Host Collection; verbatimEventDate: 12-Feb-2010; **Record Level:** language: en; institutionCode: CNC; collectionCode: Insects; basisOfRecord: Pinned Specimen
- **Type status:** Other material. **Occurrence:** occurrenceDetails: http://janzen.sas.upenn.edu; catalogNumber: DHJPAR0038713; recordedBy: D.H. Janzen & W. Hallwachs; individualID: DHJPAR0038713; individualCount: 1; lifeStage: adult; preparations: pinned; otherCatalogNumbers: ASHYD2286-10,09-SRNP-68574; **Taxon:** scientificName: Cordyligaster petiolata; phylum: Arthropoda; class: Insecta; order: Diptera; family: Tachinidae; genus: Cordyligaster; specificEpithet: fuscipennis; scientificNameAuthorship: (Macquart, 1851); **Location:** continent: Central America; country: Costa Rica; countryCode: CR; stateProvince: Alajuela; county: Sector Rincon Rain Forest; locality: Area de Conservacion Guanacaste; verbatimLocality: Melas; verbatimElevation: 153; verbatimLatitude: 10.958; verbatimLongitude: -85.284; verbatimCoordinateSystem: Decimal; decimalLatitude: 10.958; decimalLongitude: -85.284; **Identification:** identifiedBy: AJ Fleming; dateIdentified: 2014; **Event:** samplingProtocol: Host Collection; verbatimEventDate: 14-Jan-2010; **Record Level:** language: en; institutionCode: CNC; collectionCode: Insects; basisOfRecord: Pinned Specimen
- **Type status:** Other material. **Occurrence:** occurrenceDetails: http://janzen.sas.upenn.edu; catalogNumber: 98-SRNP-7747; recordedBy: D.H. Janzen & W. Hallwachs; individualID: 98-SRNP-7747; individualCount: 1; lifeStage: adult; preparations: pinned; otherCatalogNumbers: 98-SRNP-7747; **Taxon:** scientificName: Cordyligaster petiolata; phylum: Arthropoda; class: Insecta; order: Diptera; family: Tachinidae; genus: Cordyligaster; specificEpithet: fuscipennis; scientificNameAuthorship: (Macquart, 1851); **Location:** continent: Central America; country: Costa Rica; countryCode: CR; stateProvince: Alajuela; county: Sector San Cristobal; locality: Area de Conservacion Guanacaste; verbatimLocality: Estacion San Cristobal; verbatimElevation: 640; verbatimLatitude: 10.87097; verbatimLongitude: -85.39144; verbatimCoordinateSystem: Decimal; decimalLatitude: 10.87097; decimalLongitude: -85.39144; **Identification:** identifiedBy: AJ Fleming; dateIdentified: 2014; **Event:** samplingProtocol: Host Collection; verbatimEventDate: 22/Sep/98; **Record Level:** language: en; institutionCode: CNC; collectionCode: Insects; basisOfRecord: Pinned Specimen
- **Type status:** Other material. **Occurrence:** occurrenceDetails: http://janzen.sas.upenn.edu; catalogNumber: 98-SRNP-7766; recordedBy: D.H. Janzen & W. Hallwachs; individualID: 98-SRNP-7766; individualCount: 1; lifeStage: adult; preparations: pinned; otherCatalogNumbers: 98-SRNP-7766; **Taxon:** scientificName: Cordyligaster petiolata; phylum: Arthropoda; class: Insecta; order: Diptera; family: Tachinidae; genus: Cordyligaster; specificEpithet: fuscipennis; scientificNameAuthorship: (Macquart, 1851); **Location:** continent: Central America; country: Costa Rica; countryCode: CR; stateProvince: Alajuela; county: Sector San Cristobal; locality: Area de Conservacion Guanacaste; verbatimLocality: Estacion San Cristobal; verbatimElevation: 640; verbatimLatitude: 10.87097; verbatimLongitude: -85.39144; verbatimCoordinateSystem: Decimal; decimalLatitude: 10.87097; decimalLongitude: -85.39144; **Identification:** identifiedBy: AJ Fleming; dateIdentified: 2014; **Event:** samplingProtocol: Host Collection; verbatimEventDate: Sep-22-1998; **Record Level:** language: en; institutionCode: CNC; collectionCode: Insects; basisOfRecord: Pinned Specimen
- **Type status:** Other material. **Occurrence:** occurrenceDetails: http://janzen.sas.upenn.edu; catalogNumber: 99-SRNP-13079; recordedBy: D.H. Janzen & W. Hallwachs; individualID: 99-SRNP-13079; individualCount: 1; lifeStage: adult; preparations: pinned; otherCatalogNumbers: 99-SRNP-13079; **Taxon:** scientificName: Cordyligaster petiolata; phylum: Arthropoda; class: Insecta; order: Diptera; family: Tachinidae; genus: Cordyligaster; specificEpithet: fuscipennis; scientificNameAuthorship: (Macquart, 1851); **Location:** continent: Central America; country: Costa Rica; countryCode: CR; stateProvince: Alajuela; county: Sector San Cristobal; locality: Area de Conservacion Guanacaste; verbatimLocality: Cementerio Viejo; verbatimElevation: 570; verbatimLatitude: 10.88111; verbatimLongitude: -85.38889; verbatimCoordinateSystem: Decimal; decimalLatitude: 10.88111; decimalLongitude: -85.38889; **Identification:** identifiedBy: AJ Fleming; dateIdentified: 2014; **Event:** samplingProtocol: Host Collection; verbatimEventDate: Sep-25-1999; **Record Level:** language: en; institutionCode: CNC; collectionCode: Insects; basisOfRecord: Pinned Specimen
- **Type status:** Other material. **Occurrence:** occurrenceDetails: http://janzen.sas.upenn.edu; catalogNumber: 99-SRNP-13070; recordedBy: D.H. Janzen & W. Hallwachs; individualID: 99-SRNP-13070; individualCount: 1; lifeStage: adult; preparations: pinned; otherCatalogNumbers: 99-SRNP-13070; **Taxon:** scientificName: Cordyligaster petiolata; phylum: Arthropoda; class: Insecta; order: Diptera; family: Tachinidae; genus: Cordyligaster; specificEpithet: fuscipennis; scientificNameAuthorship: (Macquart, 1851); **Location:** continent: Central America; country: Costa Rica; countryCode: CR; stateProvince: Alajuela; county: Sector San Cristobal; locality: Area de Conservacion Guanacaste; verbatimLocality: Cementerio Viejo; verbatimElevation: 570; verbatimLatitude: 10.88111; verbatimLongitude: -85.38889; verbatimCoordinateSystem: Decimal; decimalLatitude: 10.88111; decimalLongitude: -85.38889; **Identification:** identifiedBy: AJ Fleming; dateIdentified: 2014; **Event:** samplingProtocol: Host Collection; verbatimEventDate: Sep-25-1999; **Record Level:** language: en; institutionCode: CNC; collectionCode: Insects; basisOfRecord: Pinned Specimen
- **Type status:** Other material. **Occurrence:** occurrenceDetails: http://janzen.sas.upenn.edu; catalogNumber: 99-SRNP-12940; recordedBy: D.H. Janzen & W. Hallwachs; individualID: 99-SRNP-12940; individualCount: 1; lifeStage: adult; preparations: pinned; otherCatalogNumbers: 99-SRNP-12940; **Taxon:** scientificName: Cordyligaster petiolata; phylum: Arthropoda; class: Insecta; order: Diptera; family: Tachinidae; genus: Cordyligaster; specificEpithet: fuscipennis; scientificNameAuthorship: (Macquart, 1851); **Location:** continent: Central America; country: Costa Rica; countryCode: CR; stateProvince: Alajuela; county: Sector San Cristobal; locality: Area de Conservacion Guanacaste; verbatimLocality: Cementerio Viejo; verbatimElevation: 570; verbatimLatitude: 10.88111; verbatimLongitude: -85.38889; verbatimCoordinateSystem: Decimal; decimalLatitude: 10.88111; decimalLongitude: -85.38889; **Identification:** identifiedBy: AJ Fleming; dateIdentified: 2014; **Event:** samplingProtocol: Host Collection; verbatimEventDate: Sep-17-1999; **Record Level:** language: en; institutionCode: CNC; collectionCode: Insects; basisOfRecord: Pinned Specimen
- **Type status:** Other material. **Occurrence:** occurrenceDetails: http://janzen.sas.upenn.edu; catalogNumber: 99-SRNP-12921; recordedBy: D.H. Janzen & W. Hallwachs; individualID: 99-SRNP-12921; individualCount: 1; lifeStage: adult; preparations: pinned; otherCatalogNumbers: 99-SRNP-12921; **Taxon:** scientificName: Cordyligaster petiolata; phylum: Arthropoda; class: Insecta; order: Diptera; family: Tachinidae; genus: Cordyligaster; specificEpithet: fuscipennis; scientificNameAuthorship: (Macquart, 1851); **Location:** continent: Central America; country: Costa Rica; countryCode: CR; stateProvince: Alajuela; county: Sector San Cristobal; locality: Area de Conservacion Guanacaste; verbatimLocality: Cementerio Viejo; verbatimElevation: 570; verbatimLatitude: 10.88111; verbatimLongitude: -85.38889; verbatimCoordinateSystem: Decimal; decimalLatitude: 10.88111; decimalLongitude: -85.38889; **Identification:** identifiedBy: AJ Fleming; dateIdentified: 2014; **Event:** samplingProtocol: Host Collection; verbatimEventDate: Sep-17-1999; **Record Level:** language: en; institutionCode: CNC; collectionCode: Insects; basisOfRecord: Pinned Specimen
- **Type status:** Other material. **Occurrence:** occurrenceDetails: http://janzen.sas.upenn.edu; catalogNumber: 99-SRNP-12923; recordedBy: D.H. Janzen & W. Hallwachs; individualID: 99-SRNP-12923; individualCount: 1; lifeStage: adult; preparations: pinned; otherCatalogNumbers: 99-SRNP-12923; **Taxon:** scientificName: Cordyligaster petiolata; phylum: Arthropoda; class: Insecta; order: Diptera; family: Tachinidae; genus: Cordyligaster; specificEpithet: fuscipennis; scientificNameAuthorship: (Macquart, 1851); **Location:** continent: Central America; country: Costa Rica; countryCode: CR; stateProvince: Alajuela; county: Sector San Cristobal; locality: Area de Conservacion Guanacaste; verbatimLocality: Cementerio Viejo; verbatimElevation: 570; verbatimLatitude: 10.88111; verbatimLongitude: -85.38889; verbatimCoordinateSystem: Decimal; decimalLatitude: 10.88111; decimalLongitude: -85.38889; **Identification:** identifiedBy: AJ Fleming; dateIdentified: 2014; **Event:** samplingProtocol: Host Collection; verbatimEventDate: Sep-17-1999; **Record Level:** language: en; institutionCode: CNC; collectionCode: Insects; basisOfRecord: Pinned Specimen
- **Type status:** Other material. **Occurrence:** occurrenceDetails: http://janzen.sas.upenn.edu; catalogNumber: 99-SRNP-12945; recordedBy: D.H. Janzen & W. Hallwachs; individualID: 99-SRNP-12945; individualCount: 1; lifeStage: adult; preparations: pinned; otherCatalogNumbers: 99-SRNP-12945; **Taxon:** scientificName: Cordyligaster petiolata; phylum: Arthropoda; class: Insecta; order: Diptera; family: Tachinidae; genus: Cordyligaster; specificEpithet: fuscipennis; scientificNameAuthorship: (Macquart, 1851); **Location:** continent: Central America; country: Costa Rica; countryCode: CR; stateProvince: Alajuela; county: Sector San Cristobal; locality: Area de Conservacion Guanacaste; verbatimLocality: Cementerio Viejo; verbatimElevation: 570; verbatimLatitude: 10.88111; verbatimLongitude: -85.38889; verbatimCoordinateSystem: Decimal; decimalLatitude: 10.88111; decimalLongitude: -85.38889; **Identification:** identifiedBy: AJ Fleming; dateIdentified: 2014; **Event:** samplingProtocol: Host Collection; verbatimEventDate: Sep-17-1999; **Record Level:** language: en; institutionCode: CNC; collectionCode: Insects; basisOfRecord: Pinned Specimen
- **Type status:** Other material. **Occurrence:** occurrenceDetails: http://janzen.sas.upenn.edu; catalogNumber: 99-SRNP-13077; recordedBy: D.H. Janzen & W. Hallwachs; individualID: 99-SRNP-13077; individualCount: 1; lifeStage: adult; preparations: pinned; otherCatalogNumbers: 99-SRNP-13077; **Taxon:** scientificName: Cordyligaster petiolata; phylum: Arthropoda; class: Insecta; order: Diptera; family: Tachinidae; genus: Cordyligaster; specificEpithet: fuscipennis; scientificNameAuthorship: (Macquart, 1851); **Location:** continent: Central America; country: Costa Rica; countryCode: CR; stateProvince: Alajuela; county: Sector San Cristobal; locality: Area de Conservacion Guanacaste; verbatimLocality: Cementerio Viejo; verbatimElevation: 570; verbatimLatitude: 10.88111; verbatimLongitude: -85.38889; verbatimCoordinateSystem: Decimal; decimalLatitude: 10.88111; decimalLongitude: -85.38889; **Identification:** identifiedBy: AJ Fleming; dateIdentified: 2014; **Event:** samplingProtocol: Host Collection; verbatimEventDate: Sep-25-1999; **Record Level:** language: en; institutionCode: CNC; collectionCode: Insects; basisOfRecord: Pinned Specimen
- **Type status:** Other material. **Occurrence:** occurrenceDetails: http://janzen.sas.upenn.edu; catalogNumber: 99-SRNP-12895; recordedBy: D.H. Janzen & W. Hallwachs; individualID: 99-SRNP-12895; individualCount: 1; lifeStage: adult; preparations: pinned; otherCatalogNumbers: 99-SRNP-12895; **Taxon:** scientificName: Cordyligaster petiolata; phylum: Arthropoda; class: Insecta; order: Diptera; family: Tachinidae; genus: Cordyligaster; specificEpithet: fuscipennis; scientificNameAuthorship: (Macquart, 1851); **Location:** continent: Central America; country: Costa Rica; countryCode: CR; stateProvince: Alajuela; county: Sector San Cristobal; locality: Area de Conservacion Guanacaste; verbatimLocality: Cementerio Viejo; verbatimElevation: 570; verbatimLatitude: 10.88111; verbatimLongitude: -85.38889; verbatimCoordinateSystem: Decimal; decimalLatitude: 10.88111; decimalLongitude: -85.38889; **Identification:** identifiedBy: AJ Fleming; dateIdentified: 2014; **Event:** samplingProtocol: Host Collection; verbatimEventDate: Sep-17-1999; **Record Level:** language: en; institutionCode: CNC; collectionCode: Insects; basisOfRecord: Pinned Specimen
- **Type status:** Other material. **Occurrence:** occurrenceDetails: http://janzen.sas.upenn.edu; catalogNumber: 99-SRNP-13071; recordedBy: D.H. Janzen & W. Hallwachs; individualID: 99-SRNP-13071; individualCount: 1; lifeStage: adult; preparations: pinned; otherCatalogNumbers: 99-SRNP-13071; **Taxon:** scientificName: Cordyligaster petiolata; phylum: Arthropoda; class: Insecta; order: Diptera; family: Tachinidae; genus: Cordyligaster; specificEpithet: fuscipennis; scientificNameAuthorship: (Macquart, 1851); **Location:** continent: Central America; country: Costa Rica; countryCode: CR; stateProvince: Alajuela; county: Sector San Cristobal; locality: Area de Conservacion Guanacaste; verbatimLocality: Cementerio Viejo; verbatimElevation: 570; verbatimLatitude: 10.88111; verbatimLongitude: -85.38889; verbatimCoordinateSystem: Decimal; decimalLatitude: 10.88111; decimalLongitude: -85.38889; **Identification:** identifiedBy: AJ Fleming; dateIdentified: 2014; **Event:** samplingProtocol: Host Collection; verbatimEventDate: Sep-25-1999; **Record Level:** language: en; institutionCode: CNC; collectionCode: Insects; basisOfRecord: Pinned Specimen
- **Type status:** Other material. **Occurrence:** occurrenceDetails: http://janzen.sas.upenn.edu; catalogNumber: 01-SRNP-4155; recordedBy: D.H. Janzen & W. Hallwachs; individualID: 01-SRNP-4155; individualCount: 1; lifeStage: adult; preparations: pinned; otherCatalogNumbers: 01-SRNP-4155; **Taxon:** scientificName: Cordyligaster petiolata; phylum: Arthropoda; class: Insecta; order: Diptera; family: Tachinidae; genus: Cordyligaster; specificEpithet: fuscipennis; scientificNameAuthorship: (Macquart, 1851); **Location:** continent: Central America; country: Costa Rica; countryCode: CR; stateProvince: Alajuela; county: Sector Rincon Rain Forest; locality: Area de Conservacion Guanacaste; verbatimLocality: Camino Rio Francia; verbatimElevation: 410; verbatimLatitude: 10.90425; verbatimLongitude: -85.28651; verbatimCoordinateSystem: Decimal; decimalLatitude: 10.90425; decimalLongitude: -85.28651; **Identification:** identifiedBy: AJ Fleming; dateIdentified: 2014; **Event:** samplingProtocol: Host Collection; verbatimEventDate: Jan-27-2001; **Record Level:** language: en; institutionCode: CNC; collectionCode: Insects; basisOfRecord: Pinned Specimen
- **Type status:** Other material. **Occurrence:** occurrenceDetails: http://janzen.sas.upenn.edu; catalogNumber: 01-SRNP-4156; recordedBy: D.H. Janzen & W. Hallwachs; individualID: 01-SRNP-4156; individualCount: 1; lifeStage: adult; preparations: pinned; otherCatalogNumbers: 01-SRNP-4156; **Taxon:** scientificName: Cordyligaster petiolata; phylum: Arthropoda; class: Insecta; order: Diptera; family: Tachinidae; genus: Cordyligaster; specificEpithet: fuscipennis; scientificNameAuthorship: (Macquart, 1851); **Location:** continent: Central America; country: Costa Rica; countryCode: CR; stateProvince: Alajuela; county: Sector Rincon Rain Forest; locality: Area de Conservacion Guanacaste; verbatimLocality: Camino Rio Francia; verbatimElevation: 410; verbatimLatitude: 10.90425; verbatimLongitude: -85.28651; verbatimCoordinateSystem: Decimal; decimalLatitude: 10.90425; decimalLongitude: -85.28651; **Identification:** identifiedBy: AJ Fleming; dateIdentified: 2014; **Event:** samplingProtocol: Host Collection; verbatimEventDate: Jan-27-2001; **Record Level:** language: en; institutionCode: CNC; collectionCode: Insects; basisOfRecord: Pinned Specimen
- **Type status:** Other material. **Occurrence:** occurrenceDetails: http://janzen.sas.upenn.edu; catalogNumber: 04-SRNP-42944; recordedBy: D.H. Janzen & W. Hallwachs; individualID: 04-SRNP-42944; individualCount: 1; lifeStage: adult; preparations: pinned; otherCatalogNumbers: 04-SRNP-42944; **Taxon:** scientificName: Cordyligaster petiolata; phylum: Arthropoda; class: Insecta; order: Diptera; family: Tachinidae; genus: Cordyligaster; specificEpithet: fuscipennis; scientificNameAuthorship: (Macquart, 1851); **Location:** continent: Central America; country: Costa Rica; countryCode: CR; stateProvince: Alajuela; county: Sector Rincon Rain Forest; locality: Area de Conservacion Guanacaste; verbatimLocality: Sendero Parcelas; verbatimElevation: 375; verbatimLatitude: 10.90777; verbatimLongitude: -85.29137; verbatimCoordinateSystem: Decimal; decimalLatitude: 10.90777; decimalLongitude: -85.29137; **Identification:** identifiedBy: AJ Fleming; dateIdentified: 2014; **Event:** samplingProtocol: Host Collection; verbatimEventDate: Dec-13-2004; **Record Level:** language: en; institutionCode: CNC; collectionCode: Insects; basisOfRecord: Pinned Specimen
- **Type status:** Other material. **Occurrence:** occurrenceDetails: http://janzen.sas.upenn.edu; catalogNumber: 04-SRNP-42943; recordedBy: D.H. Janzen & W. Hallwachs; individualID: 04-SRNP-42943; individualCount: 1; lifeStage: adult; preparations: pinned; otherCatalogNumbers: 04-SRNP-42943; **Taxon:** scientificName: Cordyligaster petiolata; phylum: Arthropoda; class: Insecta; order: Diptera; family: Tachinidae; genus: Cordyligaster; specificEpithet: fuscipennis; scientificNameAuthorship: (Macquart, 1851); **Location:** continent: Central America; country: Costa Rica; countryCode: CR; stateProvince: Alajuela; county: Sector Rincon Rain Forest; locality: Area de Conservacion Guanacaste; verbatimLocality: Sendero Parcelas; verbatimElevation: 375; verbatimLatitude: 10.90777; verbatimLongitude: -85.29137; verbatimCoordinateSystem: Decimal; decimalLatitude: 10.90777; decimalLongitude: -85.29137; **Identification:** identifiedBy: AJ Fleming; dateIdentified: 2014; **Event:** samplingProtocol: Host Collection; verbatimEventDate: Dec-13-2004; **Record Level:** language: en; institutionCode: CNC; collectionCode: Insects; basisOfRecord: Pinned Specimen

#### Description

Male (Fig. 5); Head: fronto orbital plate with silver tomentosity except at apex near ocellar triangle where silver gives way to black; antenna black with plumose arista; eye bare; ocellar bristles parallel and proclinate approximately twice the length of the ocellar triangle; fronto-orbital plate narrowing at apex enclosing only the ocellar triangle; proclinate orbital bristles absent in males; palpus black. Thorax: dorsal surface glabrous black, very slightly tomentose when viewd laterally. 3 post-sutural supra alar bristles, (one strong anterior, second and third bristles weak; first bristle strongest, almost 2X thickness of other post sutural supra alars); apical scutellar bristles long, equal in length of subapical scutellars; subapical scutellar bristles parallel or divergent (forming a wide V); katepisternum bearing 2 bristles, lateral view of thorax bearing 3 tomentose bands apparent (apparent when viewed from the side). Wings: dark smoky black in colour, clear to slightly smoky white towards wing base; vein R_1_ haired (Fig. 4a). Legs; black. Abdomen: all black, petiolate with both discal bristles and median marginal bristles present on T1+2, T3, T4 and T5. Silver pollinosity on upper margins of abdominal segments T3, and T4. Tomentosity covering T5 but as in the case of the thorax, this is only visible when viewed in certain angles of light, in this case dorsally.

Female (Fig. 6); Head: fronto orbital plate with silver tomentosity; parafacial silver; antenna black with plumose arista; eye bare; ocellar bristles parallel and proclinate approximately twice the length of the ocellar triangle; fronto-orbital plate slightly narrowing at apex to almost the width of the ocellar triangle; frontal vitta 2 times as wide as face; proclinate orbital bristles present; palpus black. Thorax: at first glance appears glabrous black, however under certain angles of light a very light tomentum becomes apparent. Three post-sutural supra alar bristles, (two strong anterior, and third one weak; second bristle strongest, 1.5X thickness of first pssa; apical scutellar bristles long, equal in length of subapical scutellars; scutellar bristles divergent (forming a wide V); katepisternum bearing 2 bristles, tomentose bands as in male, these bands apparent when viewed laterally. Wings: smoky black in colour, dark amber towards wing base; vein R1 haired, vein R_3_ haired along ½ of its length. Legs: black. Abdomen:  all black, petiolate petiolate with both discal bristles and median marginal bristles present on T1+2, T3, T4 and T5. Silver pollinosity on upper margins of abdominal segments T3, and T4. Very light tomentosity present on T5 but as in the case of the thorax, this is only visible under certain angles of light.

#### Diagnosis

This species is easily recognized by its relatively large size, black palpus, black antenna, and all black abdomen. It is distinguished from *C. petiolata* (Wiedemann 1830), by the lack of yellow spots on T3 (Sabrosky 1973). Species has the DNA barcode recorded below:

AACTTTATACTTTATTTTCGGTGCTTGATCAGGAATACTAGGAACATCTTTAAGAATTTTAATTCGAACAGAATTAGGACATCCAGGTTCACTAATTGGAGATGATCAAATTTATAACGTAATTGTAACAGCTCATGCTTTTATTATAATTTTTTTTATAGTTATACCAATTATAATTGGAGGATTTGGAAATTGATTAGTTCCTTTAATATTAGGAGCTCCAGATATAGCTTTTCCTCGAATAAATAATATAAGATTTTGACTACTTCCCCCTTCTTTATTACTTCTCCTAATTGGTAGAATAGTTGAAAATGGAGCTGGAACAGGATGAACAGTTTACCCTCCTTTATCTTCTAATATTGCACATAGAGGATCTTCTGTTGACTTAACTATTTTTTCACTACATTTAGCAGGTATTTCTTCTATTATAGGAGCTGTAAATTTTATTACAACAGTAATTAATATACGATCAACAGGAATTACATTTGATCGAATACCTTTATTTGTTTGATCTGTAGCAATTACAGCATTATTATTACTTTTATCTTTACCTGTATTAGCAGGAGCTATTACCATATTATTAACTGATCGAAATATAAATACTTCTTTTTTTGACCCAGCAGGAGGAGGAGANCCTATTTTATACCAACATTTATTT

Genetic comparison to the type specimens of previously know species was outside the scope of this paper, however the authors have selected to give the barcode data here as a diagnostic character such that it is readily available for future works which may undertake the barcoding of those previously described types.

#### Distribution

Costa Rica, ACG, Prov. Guanacaste, rain forest, 153 – 640m elevation. Originally described from "South America", this species has been found to be very widely distributed, from Brazil, west to Bolivia and Peru, and North to Guatemala.

#### Ecology

**Hosts:** Three species of leaf-rolling spilomeline Crambidae feeding on leaves of rain forest Urticaceae.

## Identification Keys

### Revised key to the species of *Cordyligaster* Macquart

**Table:** 

1	Calyptere reduced to mere rounded rims	2
–	Calyptere normal in size	6
2	Thorax yellow on sides, only mesonotum largely black; antenna entirely yellowish; all coxae, femora, and tibiae yellow	*C. analis* (Macquart)
–	Thorax black; at least third antennal segment black; legs black except in *tipuliformis*	3
3	Legs chiefly yellowish, including fore coxae, femora more or less infuscated centrally; palpi yellow	*C. tipuliformis* Walker
–	Legs black; palpi black	4
4	Terga1+2, T3, and base of T4 yellow except for narrow black dorsal vitta	*C. townsendi* Guimaraes
–	Abdomen predominantly shining black, at most narrowly yellow at base of T3 (apparent T1+2), and with usual silvery tomentose bands at bases of T3 and T4	5
5	Disk of mesonotum brilliantly shining; T3 with a pair of yellow spots at base, in ground color beneath band of tomentum	*C. petiolata* (Wiedemann)
–	Mesonotum thinly tomentose; T3 black in ground color, without yellow spots beneath band of tomentum	*C. fuscipennis* (Macquart)
6	Thorax covered with golden pollinosity, with faint dark stripes; antenna dark red; palpi yellow	*C. nyomala* Townsend
–	Thorax with dark ground colour and no golden pollen; antenna either entirely black or only pedicel reddish	7
7	Thorax with faintly visible silver pollinosity forming the appearance of visible dark vittae; crossvein dm-cu undulated appearing vaguely S shaped; pedicel and palpus appearing rusty brown almost orange; only posterior katepisternal bristle present; wings smoky reddish/brown	*C. minuscula* Wulp
–	Thorax glabrous black with very little silver pollinosity; crossvein dm-cu not undulated; vein R_4+5 _haired up to crosvein r-m; pedicel and palpus black; 2 katepisternal bristle present; wings smoky yellow	*C. capellii* **n. sp.**

Key adapted from Sabrosky 1973 to include all species presently occuring within the genus.

## Analysis

Mitochondrial DNA barcodes from the two species of *Cordyligaster* displayed no heteroplasmy or double banding that might suggest the inadvertent amplification and sequencing of a nuclear pseudogene.  Sequences displayed the characteristic AT bias of insect mitochondrial DNA (70%) and showed very little intraspecific variation within their DNA barcode region (min 0.05%, min 0.31% respectively) with greater inter-specific variation evident between them (6.83%).

## Supplementary Material

Supplementary material 1 Cordyligaster NJ 7Oct14 Data type: Neighbor Joining Tree
File: oo_32773.pdf DHJ, WH

Supplementary material 2 Cordyligaster exel 7Oct14 Data type: Excel spreadsheet displaying relevant data from Neighbor Joining tree
File: oo_32774.xls DHJ, WH

XML Treatment for Cordyligaster capellii

XML Treatment for Cordyligaster fuscipennis

## Figures and Tables

**Figure 1a.**
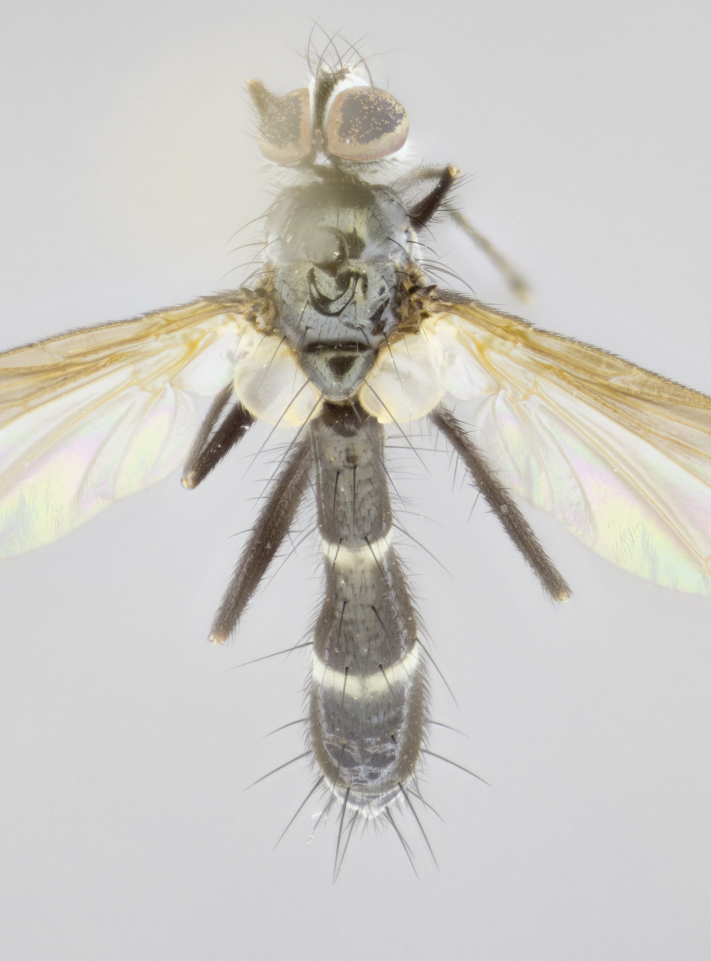
Dorsal view of *Cordyligaster capellii* male

**Figure 1b.**
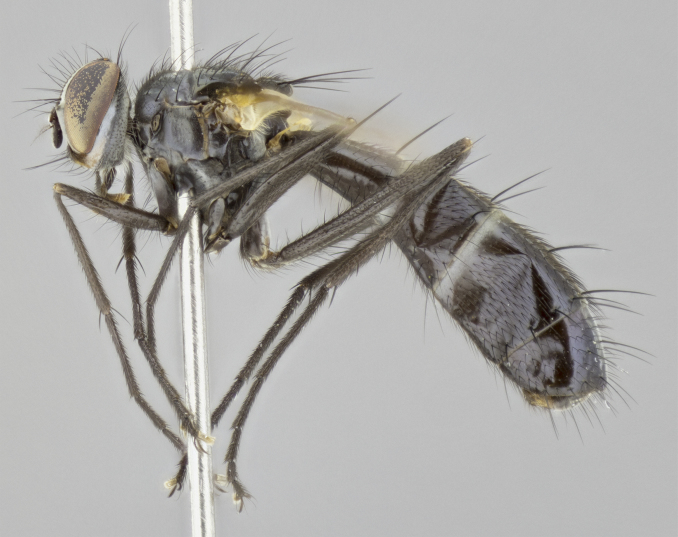
Lateral view of *Cordyligaster capellii* male

**Figure 1c.**
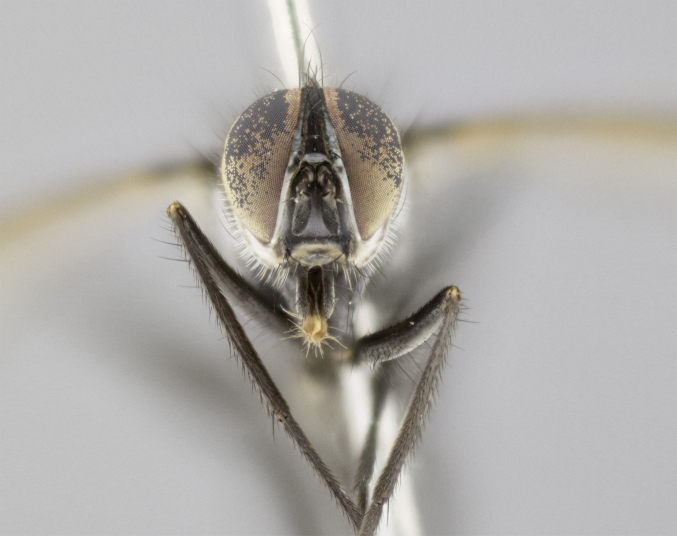
Frontal view of *Cordyligaster capellii* male head

**Figure 1d.**
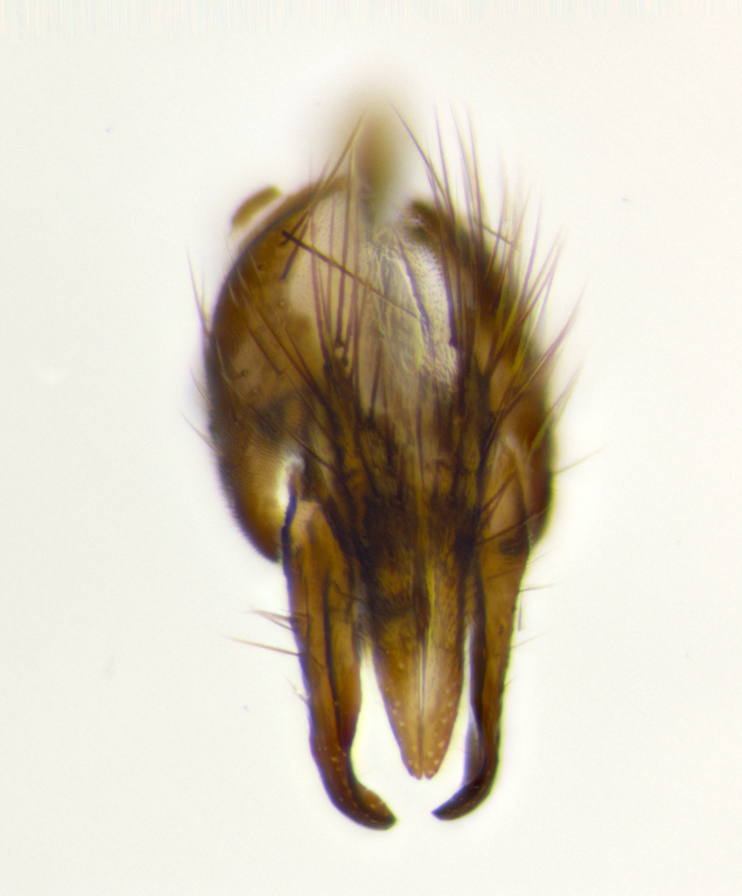
Dorsal view of male terminalia

**Figure 1e.**
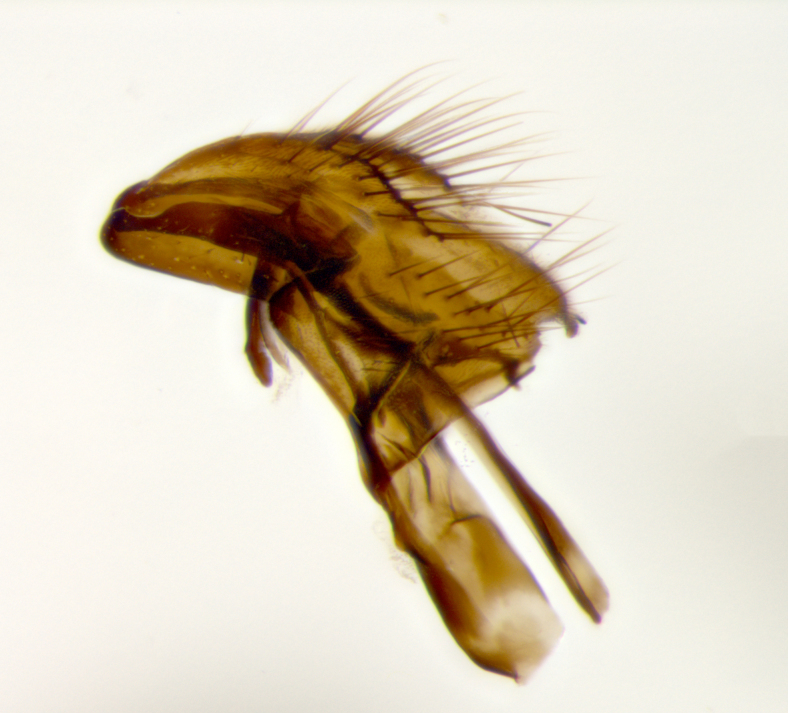
Lateral view of male terminalia

**Figure 2a.**
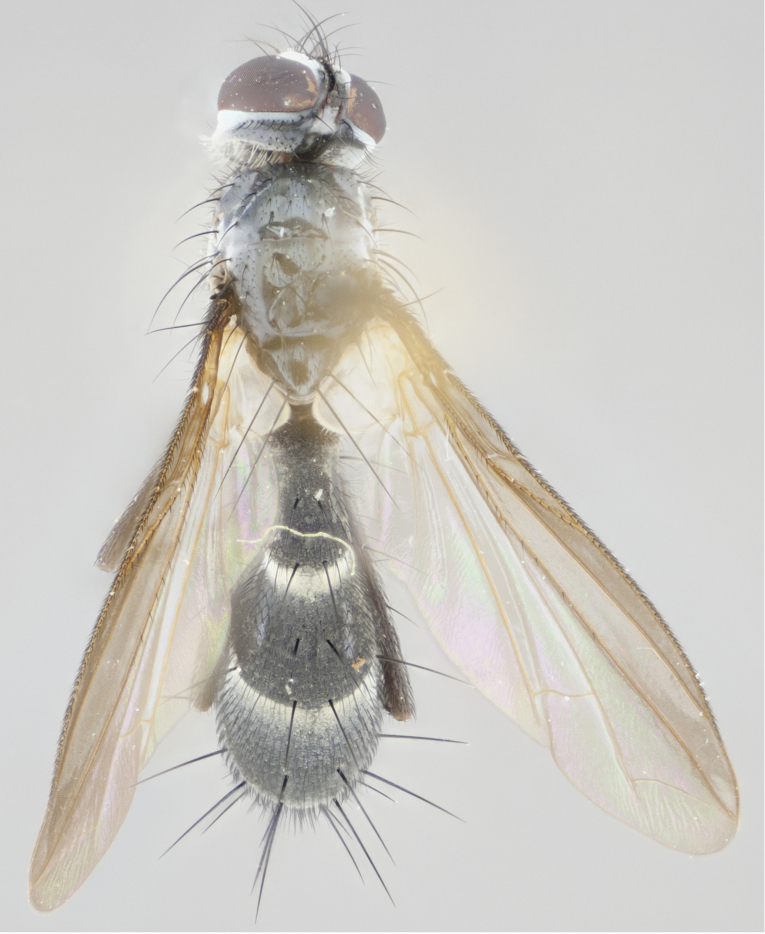
Dorsal view of *Cordyligaster capellii* female

**Figure 2b.**
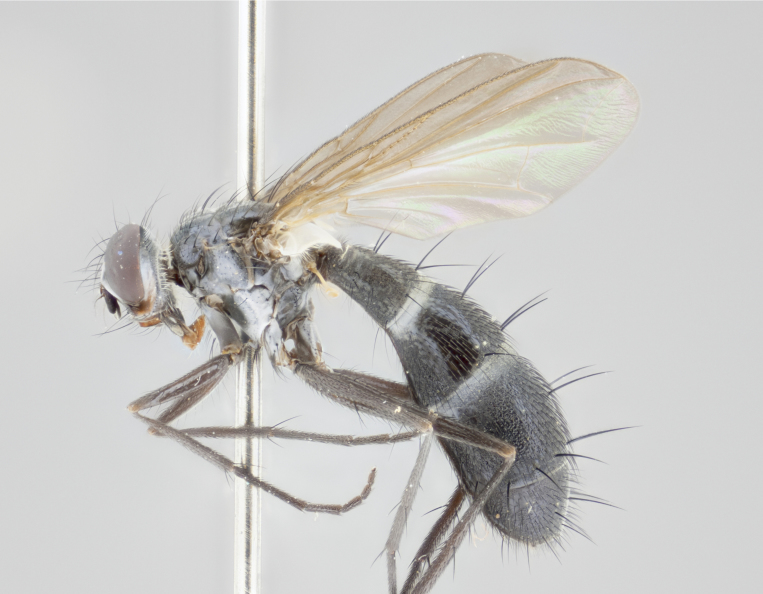
Lateral view of *Cordyligaster capellii* female

**Figure 2c.**
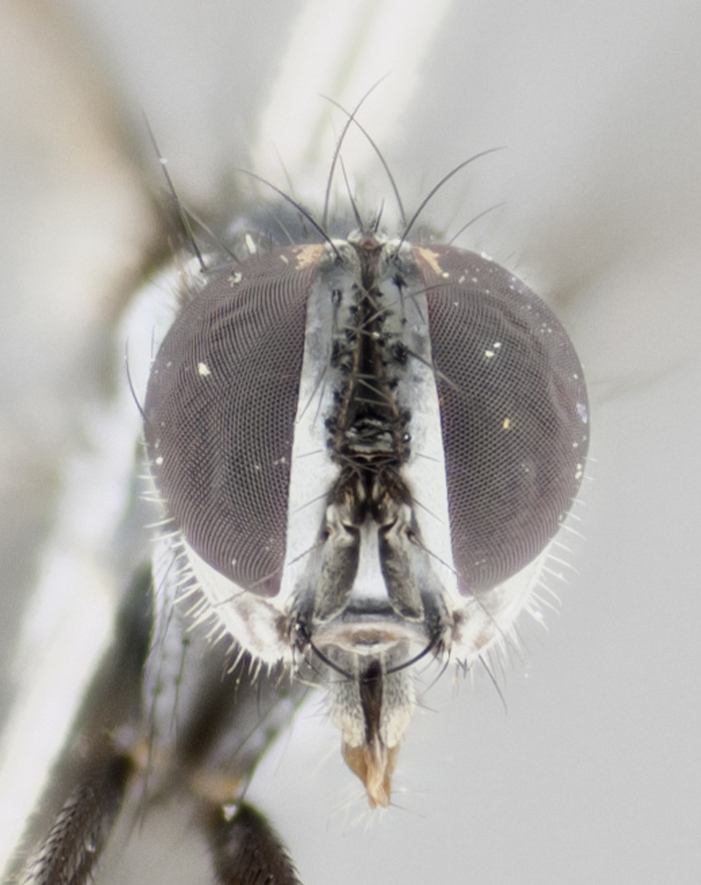
Frontal view of head of *Cordyligaster capellii* female

**Figure 3a.**
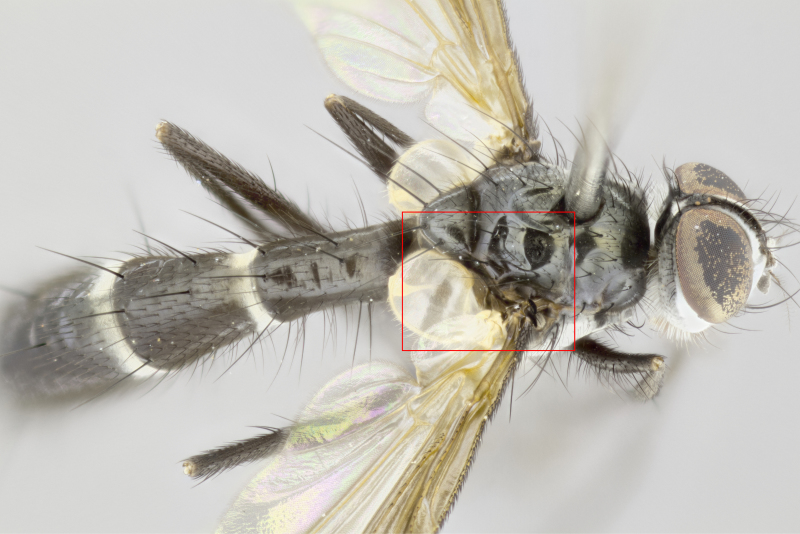


**Figure 3b.**
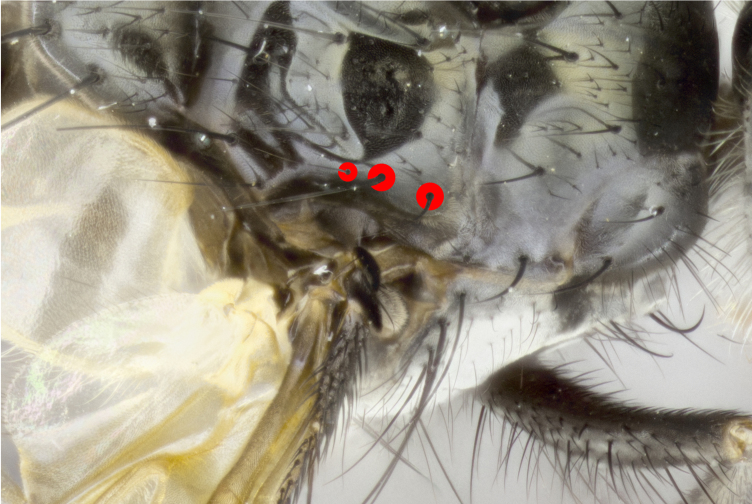


**Figure 4a.**
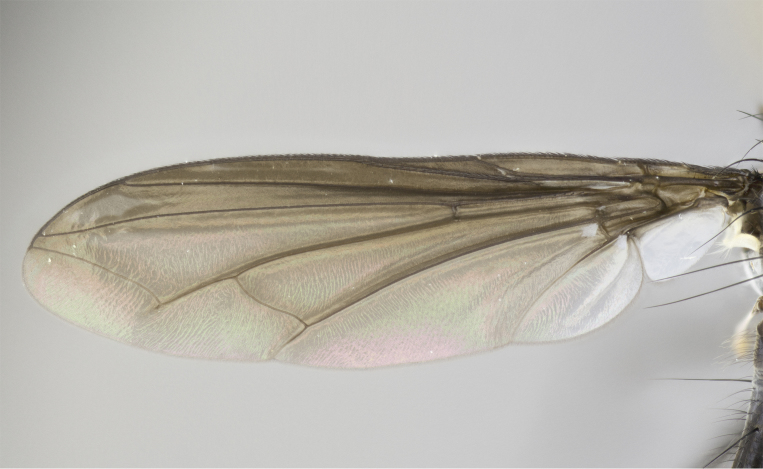
*Cordyligaster fuscipennis*

**Figure 4b.**
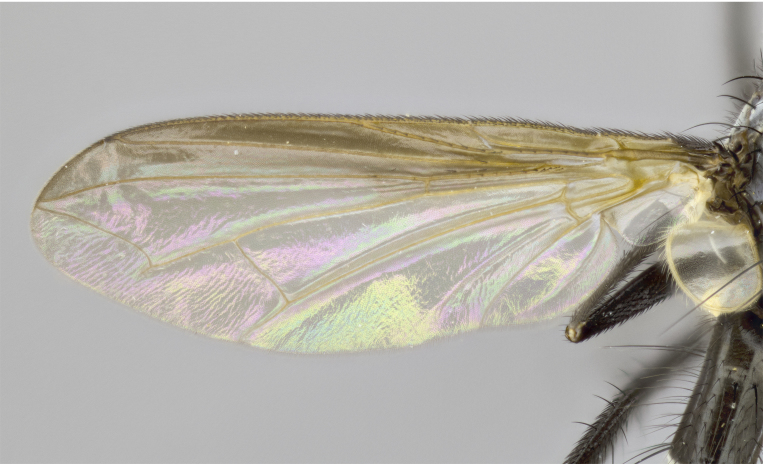
*Cordyligaster capellii*

**Figure 4c.**
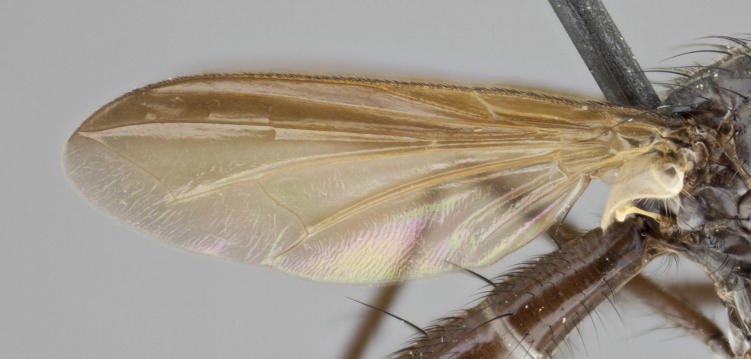
*Cordyligaster minuscula*

**Figure 5a.**
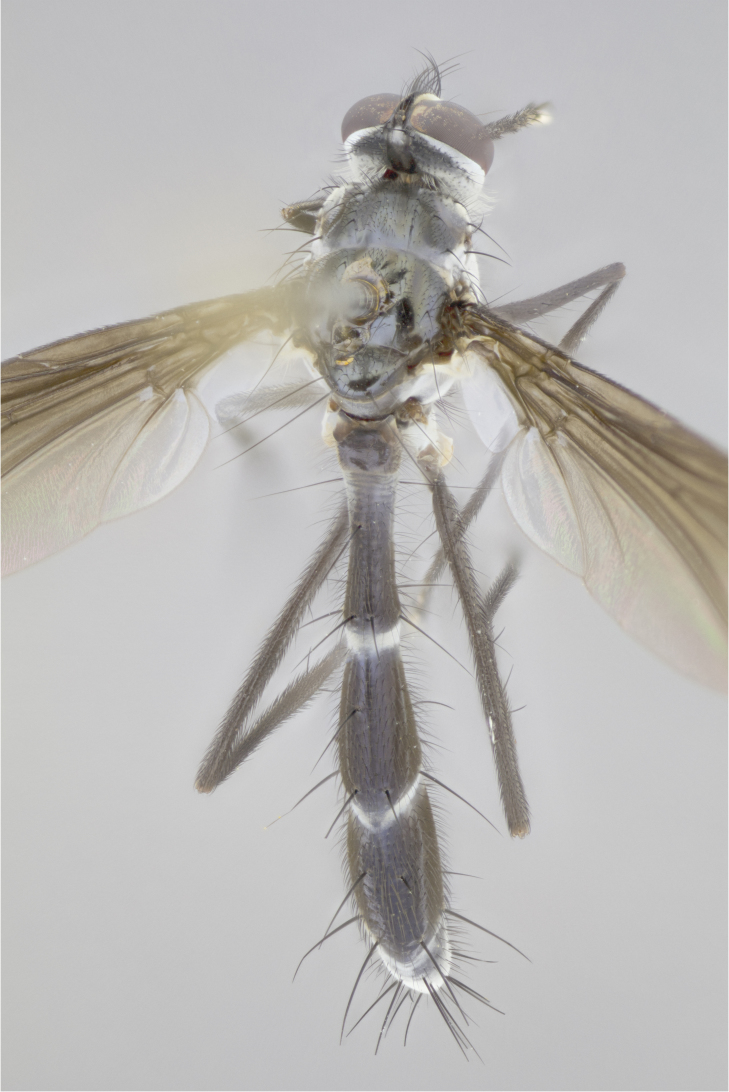
Dorsal view of *Cordyligaster fuscipennis* male

**Figure 5b.**
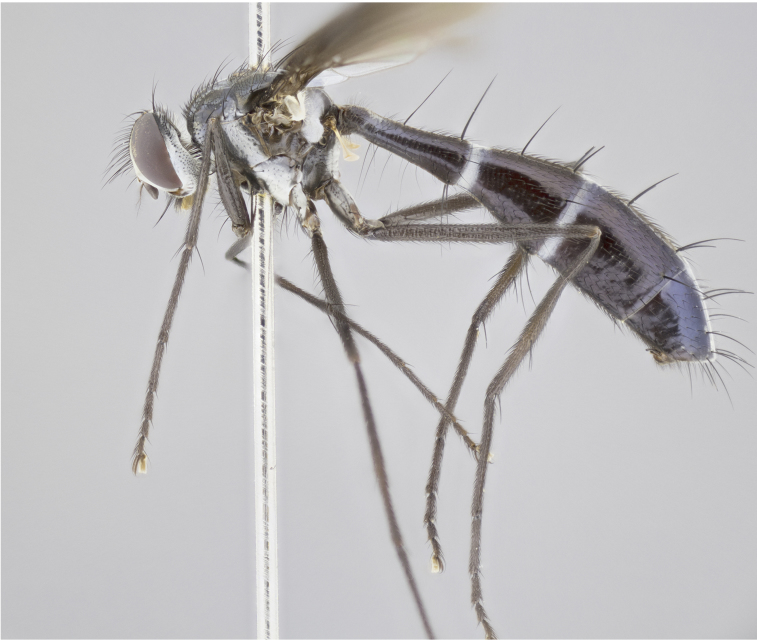
Lateral view of *Cordyligaster fuscipennis* male

**Figure 5c.**
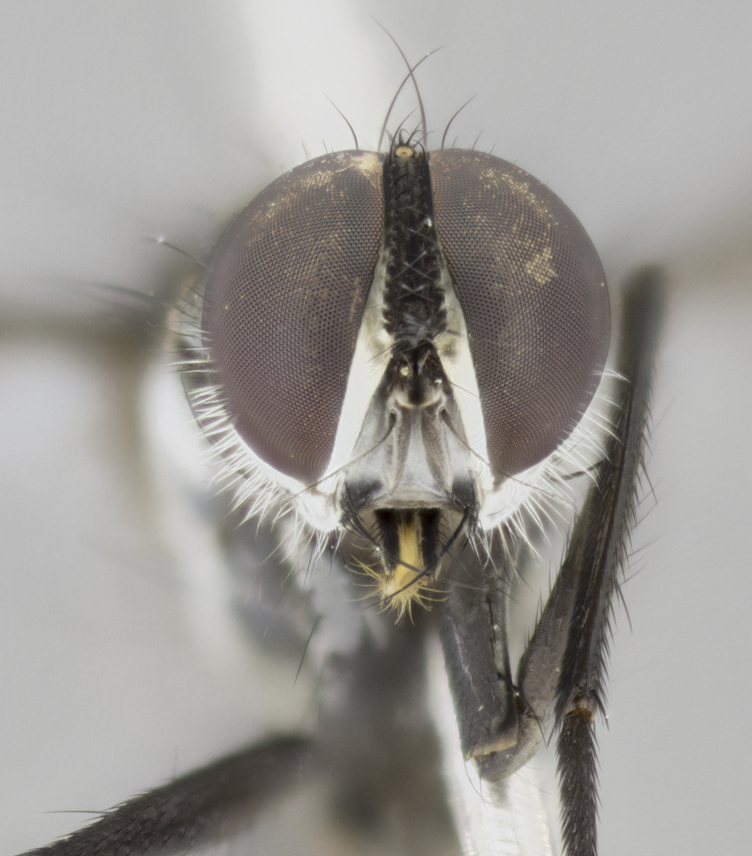
Frontal view of head of *Cordyligaster fuscipennis* male

**Figure 6a.**
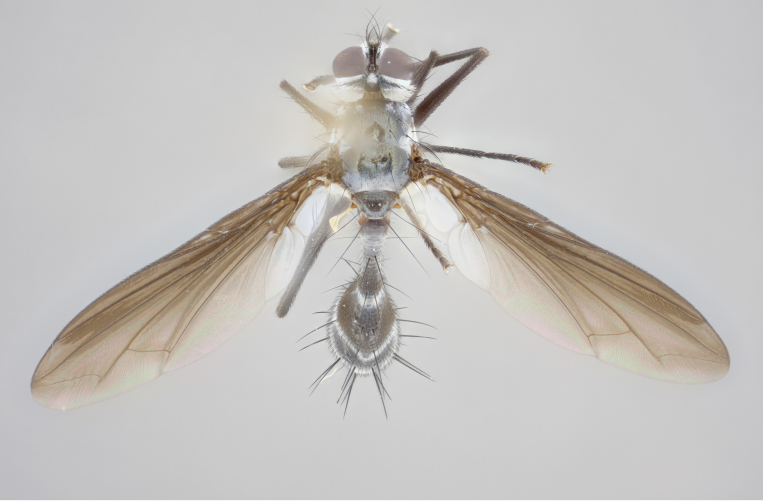
Dorsal view of *Cordyligaster fuscipennis* female

**Figure 6b.**
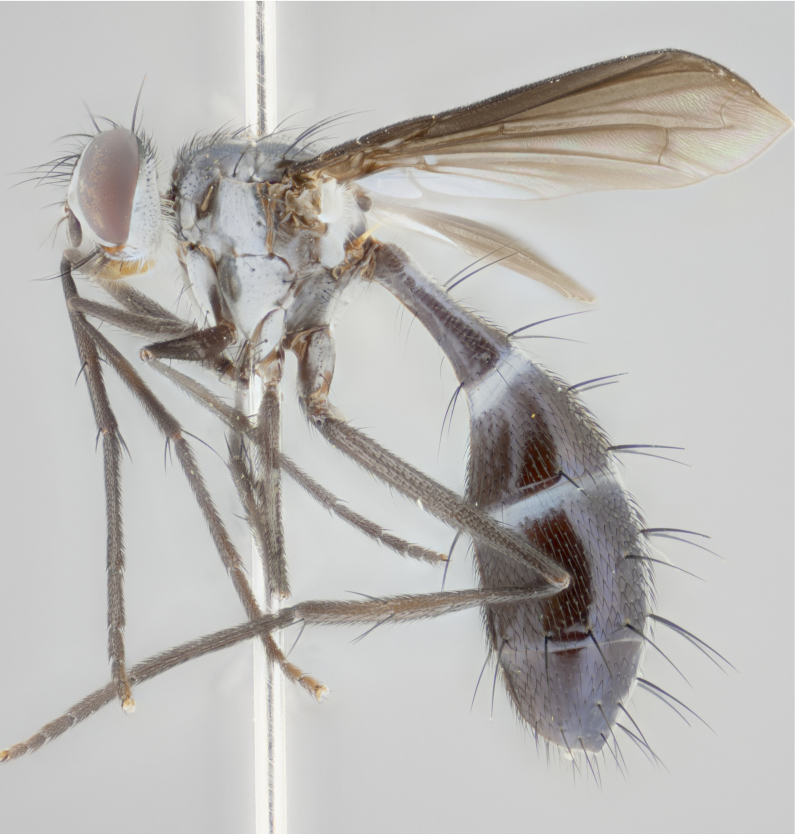
Lateral view of *Cordyligaster fuscipennis* female

**Figure 6c.**
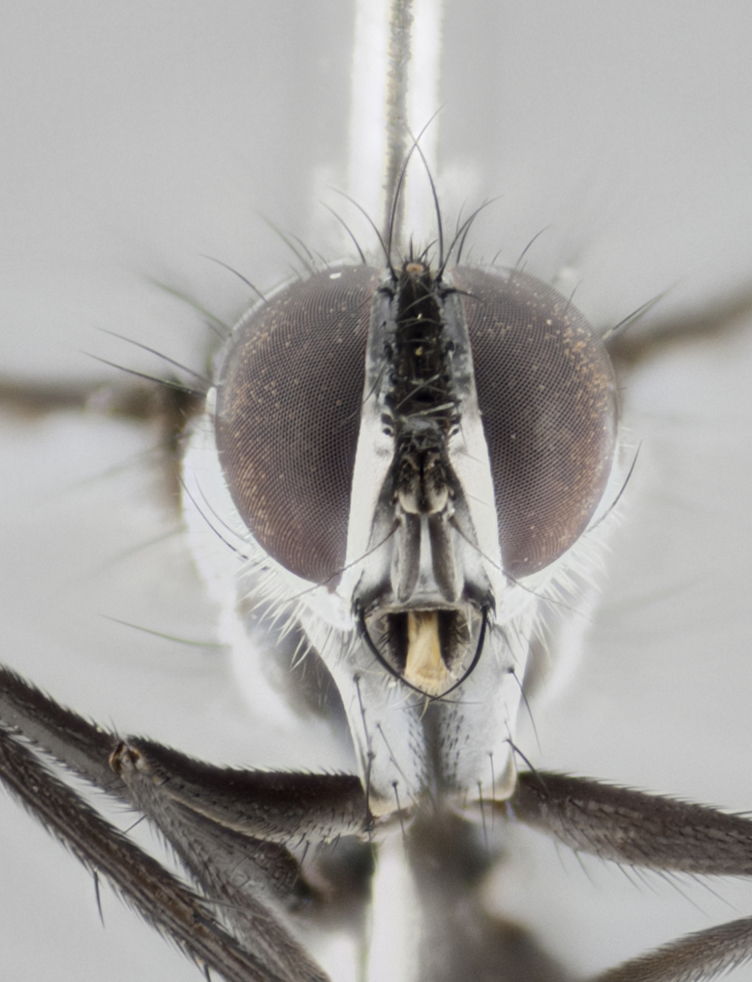
Frontal view of head of *Cordyligaster fuscipennis* female
