# Supplementary material for: A new species of Cordyligaster Macquart, reared from caterpillars in Area de Conservacion Guanacaste, northwestern Costa Rica
Source: Biodivers Data J. 2014 Nov 24;(2):e4174. doi: 10.3897/BDJ.2.e4174 (PMC4266961; doi:10.3897/BDJ.2.e4174)
Supplement: Supplementary material 1 — Cordyligaster NJ 7Oct14 [file biodiversity_data_journal-2-e4174-s001.pdf]

BOLD TaxonID Tree

```
Title            : SEARCH: Marker(COI-5P), Sample ids(41 ids) [SEARCH1]
Date             : 7-October-2014
Data Type        : Nucleotide
Distance Model   : Kimura 2 Parameter
Marker           : COI-5P
Codon Positions  : 1st, 2nd, 3rd
Labels           : Extra Info, SampleID, Sequence Length
Filters          : Length > 200
Colorization     : [blue]=Stop Codons [red]=Contamination or misidentification
Attachment       : Photographs & Spreadsheet

Sequence Count   : 41
Species count    : 3
Genus count      : 1
Family count     : 1
Unidentified     : 0
```

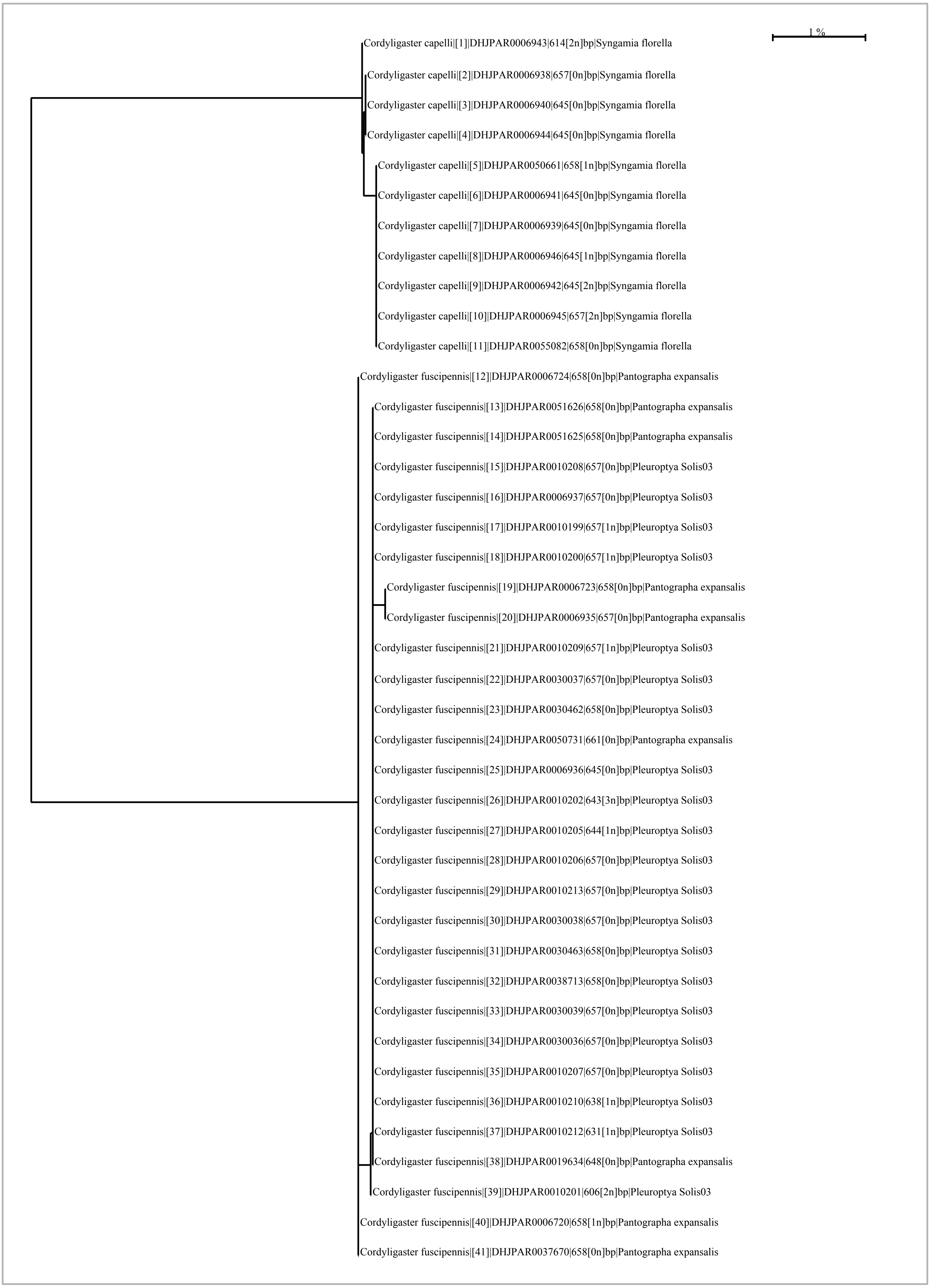
1 %
Cordyligaster capelli|[1]|DHJPAR0006943|614[2n]bp|Syngamia florella
Cordyligaster capelli|[2]|DHJPAR0006938|657[0n]bp|Syngamia florella
Cordyligaster capelli|[3]|DHJPAR0006940|645[0n]bp|Syngamia florella
Cordyligaster capelli|[4]|DHJPAR0006944|645[0n]bp|Syngamia florella
Cordyligaster capelli|[5]|DHJPAR0050661|658[1n]bp|Syngamia florella
Cordyligaster capelli|[6]|DHJPAR0006941|645[0n]bp|Syngamia florella
Cordyligaster capelli|[7]|DHJPAR0006939|645[0n]bp|Syngamia florella
Cordyligaster capelli|[8]|DHJPAR0006946|645[1n]bp|Syngamia florella
Cordyligaster capelli|[9]|DHJPAR0006942|645[2n]bp|Syngamia florella
Cordyligaster capelli|[10]|DHJPAR0006945|657[2n]bp|Syngamia florella
Cordyligaster capelli|[11]|DHJPAR0055082|658[0n]bp|Syngamia florella
Cordyligaster fuscipennis|[12]|DHJPAR0006724|658[0n]bp|Pantographa expansalis
Cordyligaster fuscipennis|[13]|DHJPAR0051626|658[0n]bp|Pantographa expansalis
Cordyligaster fuscipennis|[14]|DHJPAR0051625|658[0n]bp|Pantographa expansalis
Cordyligaster fuscipennis|[15]|DHJPAR0010208|657[0n]bp|Pleuroptya Solis03
Cordyligaster fuscipennis|[16]|DHJPAR0006937|657[0n]bp|Pleuroptya Solis03
Cordyligaster fuscipennis|[17]|DHJPAR0010199|657[1n]bp|Pleuroptya Solis03
Cordyligaster fuscipennis|[18]|DHJPAR0010200|657[1n]bp|Pleuroptya Solis03
Cordyligaster fuscipennis|[19]|DHJPAR0006723|658[0n]bp|Pantographa expansalis
Cordyligaster fuscipennis|[20]|DHJPAR0006935|657[0n]bp|Pantographa expansalis
Cordyligaster fuscipennis|[21]|DHJPAR0010209|657[1n]bp|Pleuroptya Solis03
Cordyligaster fuscipennis|[22]|DHJPAR0030037|657[0n]bp|Pleuroptya Solis03
Cordyligaster fuscipennis|[23]|DHJPAR0030462|658[0n]bp|Pleuroptya Solis03
Cordyligaster fuscipennis|[24]|DHJPAR0050731|661[0n]bp|Pantographa expansalis
Cordyligaster fuscipennis|[25]|DHJPAR0006936|645[0n]bp|Pleuroptya Solis03
Cordyligaster fuscipennis|[26]|DHJPAR0010202|643[3n]bp|Pleuroptya Solis03
Cordyligaster fuscipennis|[27]|DHJPAR0010205|644[1n]bp|Pleuroptya Solis03
Cordyligaster fuscipennis|[28]|DHJPAR0010206|657[0n]bp|Pleuroptya Solis03
Cordyligaster fuscipennis|[29]|DHJPAR0010213|657[0n]bp|Pleuroptya Solis03
Cordyligaster fuscipennis|[30]|DHJPAR0030038|657[0n]bp|Pleuroptya Solis03
Cordyligaster fuscipennis|[31]|DHJPAR0030463|658[0n]bp|Pleuroptya Solis03
Cordyligaster fuscipennis|[32]|DHJPAR0038713|658[0n]bp|Pleuroptya Solis03
Cordyligaster fuscipennis|[33]|DHJPAR0030039|657[0n]bp|Pleuroptya Solis03
Cordyligaster fuscipennis|[34]|DHJPAR0030036|657[0n]bp|Pleuroptya Solis03
Cordyligaster fuscipennis|[35]|DHJPAR0010207|657[0n]bp|Pleuroptya Solis03
Cordyligaster fuscipennis|[36]|DHJPAR0010210|638[1n]bp|Pleuroptya Solis03
Cordyligaster fuscipennis|[37]|DHJPAR0010212|631[1n]bp|Pleuroptya Solis03
Cordyligaster fuscipennis|[38]|DHJPAR0019634|648[0n]bp|Pantographa expansalis
Cordyligaster fuscipennis|[39]|DHJPAR0010201|606[2n]bp|Pleuroptya Solis03
Cordyligaster fuscipennis|[40]|DHJPAR0006720|658[1n]bp|Pantographa expansalis
Cordyligaster fuscipennis|[41]|DHJPAR0037670|658[0n]bp|Pantographa expansalis
